# Expanding the fluorescence toolkit: molecular design, synthesis and biological application of unnatural amino acids

**DOI:** 10.1039/d5sc05745k

**Published:** 2025-08-25

**Authors:** Olivia Marshall, Andrew Sutherland

**Affiliations:** a School of Chemistry, University of Glasgow Joseph Black Building, University Avenue Glasgow G12 8QQ UK Andrew.Sutherland@glasgow.ac.uk

## Abstract

Fluorescence imaging has become an indispensable tool in modern biology, enabling the visualisation of dynamic molecular processes with spatial and temporal precision. Traditional strategies rely heavily on the conjugation of large, extrinsic fluorophores, such as green fluorscent protein or organic dyes, through linkers to proteins or peptides of interest. While sometimes effective, these bulky labels can interfere with native protein structure, function, and interactions, limiting their utility in studying sensitive or compact biological systems. In contrast, fluorescent unnatural amino acids that can be site-specifically incorporated into proteins with minimal perturbation, provide high-resolution insights into molecular behaviour while preserving biological integrity. This perspective focuses on recent advances in the synthesis and application of fluorescent unnatural amino acids, particularly through the structural modification of natural aromatic-containing residues such as phenylalanine, tyrosine, and tryptophan. Additionally, the incorporation of small-molecule fluorophores as amino acid side chains to create versatile probes is also discussed. The utility of these fluorescent amino acids in biological chemistry is highlighted through their application in imaging protein dynamics, measuring enzyme activity and studying biomolecular interactions. Together, these advances establish fluorescent amino acids as a valuable and evolving toolkit for minimally invasive biological imaging.

## Abstract 2

This perspective describes recent advances in the discovery of fluorescent unnatural amino acids as intrinsic peptidic probes for the minimally invasive measurement, sensing and imaging of biological processes.

## Graphical abstract

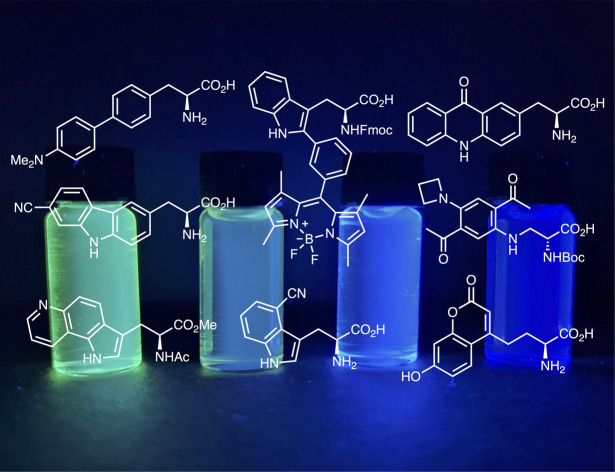

## Introduction

Fluorescence imaging is an essential technique in the life, medical and physical sciences, enabling the real-time visualisation of molecular and cellular processes with high spatial and temporal resolution.^1–5^ It has revolutionised the ability to study biological systems, from elucidating protein dynamics to mapping intracellular signalling pathways and monitoring cellular behaviour *in vivo*. However, the effectiveness of fluorescence imaging critically depends on the tools used for fluorescent labelling, and conventional strategies often suffer from significant limitations. Traditionally, proteins are labelled with large extrinsic organic fluorophores such as fluoresceins or rhodamines or attached to fluorescent proteins like green fluorescent protein (GFP).^3,6–9^ While bright with generally excellent photophysical properties, these large fluorophores can disrupt the native structure, dynamics, or function of the target proteins due to their steric bulk and chemical properties.^10^ Moreover, chemical conjugation methods used to attach these dyes such as reaction of lysine with fluorophore activated *N*-hydroxysuccinimide esters or through conjugate addition of cysteine with maleimide derivatives are often nonspecific and typically yield heterogeneous labelling, issues that are particularly problematic in sensitive live-cell or *in vivo* studies.^11^ Some studies have attempted to utilise the intrinsic fluorescence of natural amino acids, primarily l-phenylalanine (1), l-tyrosine (2) and l-tryptophan (3) to circumvent the need for external labels.^3,12^ However, the native fluorescence of these residues is inherently weak (Table 1), spectroscopically limited, and highly sensitive to environmental quenching.^13^ Tryptophan, while the most fluorescent of the three, exhibits emission in the UV range (365 nm), which is not ideal for biological imaging due to poor tissue penetration, high background autofluorescence, and potential photodamage. Phenylalanine and tyrosine have lower quantum yields and are generally impractical as fluorescent probes for most imaging applications.

**Table 1:** Chemical structures and photophysical properties of proteinogenic amino acids, l-phenylalanine (1), l-tyrosine (2) and l-tryptophan (3)^3,13^

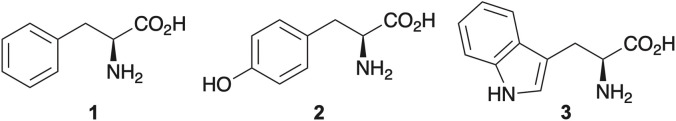					
Amino acid	*λ*_Abs_ (nm)	*ε* (cm^−1^ M^−1^)	*λ*_Em_ (nm)	*Φ*_F_	Brightness (cm^−1^ M^−1^)
l-Phe (1)	258	200	282	0.024	5
l-Tyr (2)	275	1410	310	0.14	197
l-Trp (3)	279	5580	365	0.20	1116

To overcome these challenges, the development and application of fluorescent unnatural amino acids has emerged as a powerful alternative approach.^14–16^ Fluorescent unnatural amino acids are synthetic analogues of natural amino acids that possess intrinsic fluorescence and can be incorporated site-specifically into proteins through genetic code expansion or modern solid phase peptide synthesis (SPPS) methods.^17^ Unlike large fluorophores, fluorescent unnatural amino acids are structurally similar in size to natural amino acids, which minimises steric hindrance and preserves protein structure and function. Furthermore, they offer distinct spectroscopic properties, including red-shifted excitation/emission, enhanced brightness, and environmental sensitivity, resulting in probes that are more suitable for biological imaging than their natural counterparts.^18–20^ This strategy facilitates the real-time monitoring of protein folding, conformational changes, intermolecular interactions, and subcellular localisation with high fidelity. As advancements in synthetic biology and protein engineering continue to progress, fluorescent unnatural amino acids are poised to play an increasingly central role in the development of next-generation imaging tools and molecular probes. With fluorescent unnatural amino acids emerging as powerful tools for biological imaging, this perspective highlights key synthetic developments and biological applications reported over the past decade. Emphasis is placed on strategies that modify natural l-amino acids or incorporate small-molecule fluorophores as novel side chains to expand the functional diversity of fluorescent unnatural amino acids.

## Fluorescent unnatural amino acids based on proteinogenic amino acids

### Modification of phenylalanine

Phenylalanine has significant limitations as a fluorescent imaging agent, including a low quantum yield, poor photostability, and excitation/emission wavelengths in the ultraviolet (UV) region.^12,13^ These photophysical properties not only limit sensitivity but also increase background autofluorescence and phototoxicity in biological samples, making native phenylalanine suboptimal for live-cell or *in vivo* imaging. To overcome these drawbacks, researchers have developed fluorescent unnatural amino acids derived from phenylalanine that feature extended π-conjugation systems, which red-shift excitation and emission profiles into the visible region, while also enhancing brightness and photostability.

Minimally modified phenylalanines such as cyano derivatives have been shown to possess improved photophysical properties that are more suitable for biological studies. For example, 4-cyanophenylalanine has a quantum yield that is approximately five times that of phenylalanine and can be excited at 240 nm even in the presence of tryptophan.^21^ Furthermore, the fluorescence intensity of 4-cyanophenylalanine increases significantly from aprotic to protic solvents and is pH dependent, allowing the application of this amino acid in a range of biological studies such as determining the membrane insertion kinetics of the cell-penetrating peptide TAT and monitoring amyloid formation kinetics in human islet amyloid polypeptide.^22,23^ Structural analogues such as 2- and 3-cyanophenylalanine exhibit similar photophysical properties and therefore also serve as biological probes.^24^

**Scheme 1:**
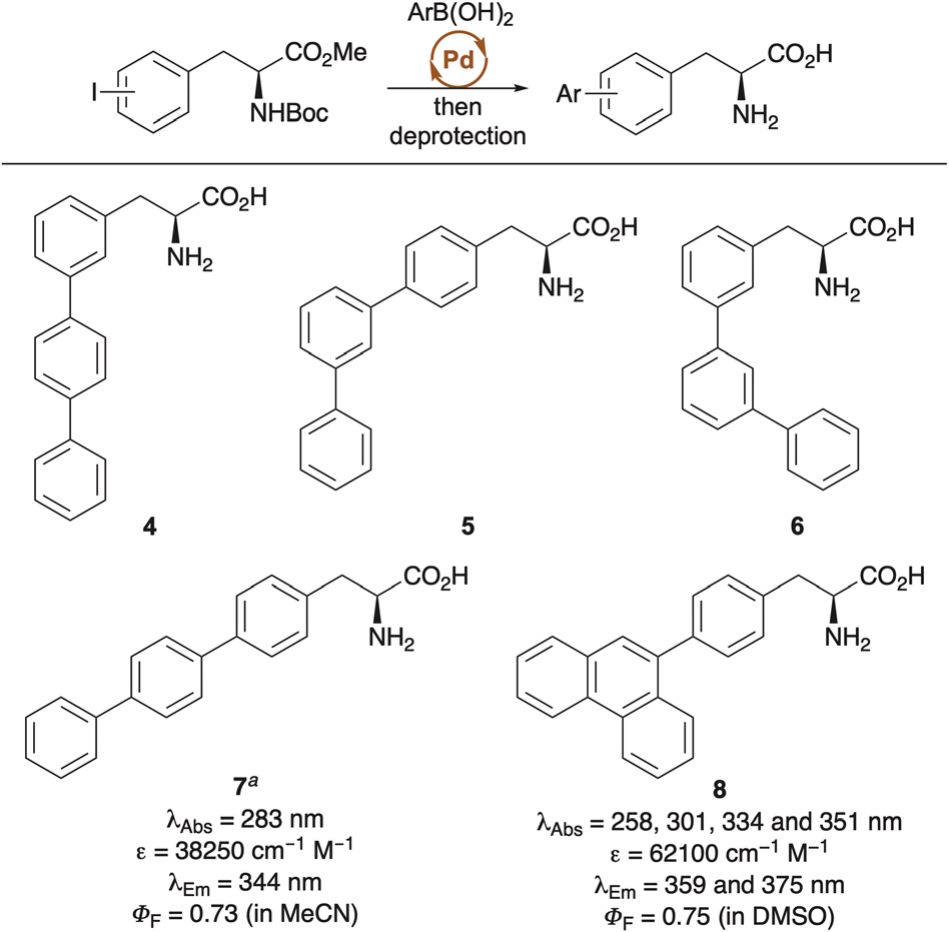
Synthesis of polyaromatic phenylalanine analogues using the Suzuki–Miyaura reaction. ^*a*^Photophysical data is for the *N*-pentenoyl derivative.

Another common modification of phenylalanine involves the incorporation of additional conjugated aromatic rings,^25–27^ typically achieved through palladium-catalysed Suzuki–Miyaura cross-coupling of halogenated phenylalanines and aryl boronic acids (Scheme 1). Benkovic, Hecht and co-workers employed this strategy to prepare a series of terphenyl amino acids (4–7), which were used to study dihydrofolate reductase (DHFR) from *E. coli*.^28,29^ Among these, linear analogue 7 exhibited the most enhanced photophysical properties relative to phenylalanine, with red-shifted absorption and emission, as well as an excellent quantum yield of 0.73. All four terphenyl amino acids were site-specifically incorporated into DHFR using an *in vitro* expression system and were employed to probe various aspects of the enzyme, such as conformational changes during inhibitor binding. A similar synthetic approach was used to prepare 4-phenanthracen-9-yl-l-phenylalanine 8, which also demonstrated an excellent quantum yield and emission in the visible region.^30^ A long absorbance tail enabled the use of this polyaromatic amino acid for fluorescent imaging of human HeLa cells upon excitation at 405 nm.

**Fig. 1:**
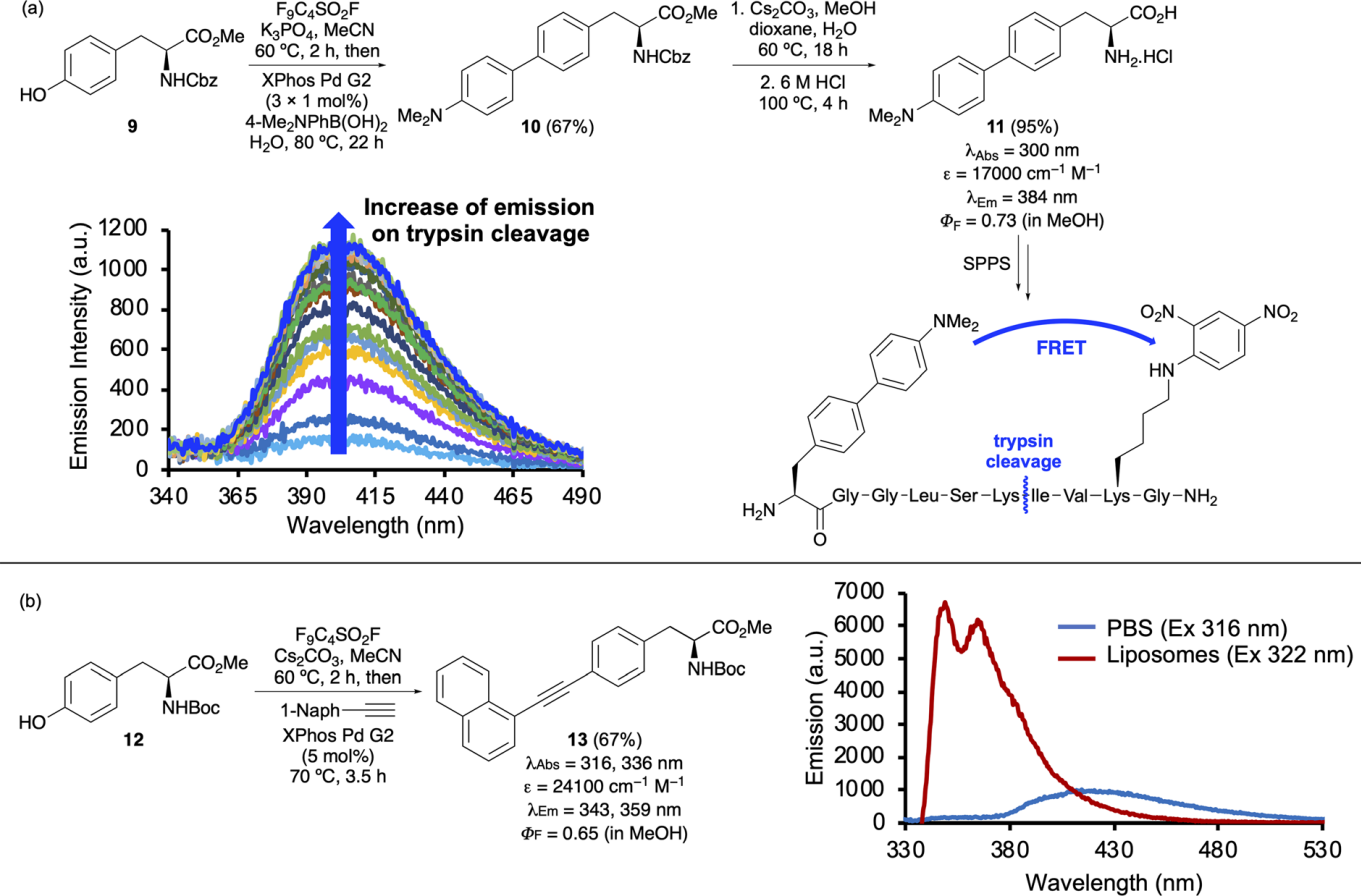
(a) One-pot synthesis of biaryl amino acid 10 and incorporation into a decapeptide for FRET measurement of trypsin cleavage. (b) One-pot synthesis of alkyne-extended phenylalanine 13 and fluorescent sensitivity for lipid-rich environments.

To generate a library of biphenyl amino acids for fluorescent probe discovery, a streamlined one-pot method was developed starting from commercially available tyrosine derivative 9.^31^ In this approach, the phenolic hydroxyl group was first activated as a nonaflate, followed by a Suzuki–Miyaura reaction with various boronic acids using the Buchwald precatalyst XPhos Pd G2.^32^ This efficient protocol allowed the synthesis of a diverse range of phenylalanine analogues and led to the discovery of dimethylaminobiphenyl amino acid 11, which exhibited strong charge transfer-based fluorescence with pronounced solvatochromic and pH sensitive properties (Fig. 1a). Notably, amino acid 11 functioned effectively as a Förster resonance energy transfer (FRET) donor for real-time monitoring of enzyme kinetics.^33^ Incorporation into an internally quenched decapeptide *via* SPPS and using 2,4-dinitrophenyllysine as a FRET acceptor resulted in suppressed emission. Upon proteolytic cleavage by trypsin, a fluorescence turn-on response was observed, allowing quantitative measurement of protease activity.

This one-pot method was also used for the synthesis of arylalkyne-extended phenylalanines (Fig. 1b).^34^ The aim of this study was to use the added conjugation of an alkyne spacer to enhance the fluorescence properties of biaryl amino acids. Similar nonaflate activation of tyrosine 12, followed by a copper-free Sonogashira cross-coupling, again using the Buchwald precatalyst XPhos Pd G2,^32^ facilitated the efficient preparation of a range of analogues with various aryl substituents. From this small library, 1-naphthyl analogue 13 emerged as the lead compound, displaying both red-shifted absorption and a high quantum yield. Comparative analysis against its biaryl analogue highlighted a 5-fold enhancement in brightness, attributable to the alkyne extension. Amino acid 13 also showed potential as a fluorescence probe for lipid-rich environments, displaying enhanced emission in artificial liposomes *versus* phosphate-buffered saline (PBS).

**Scheme 2:**
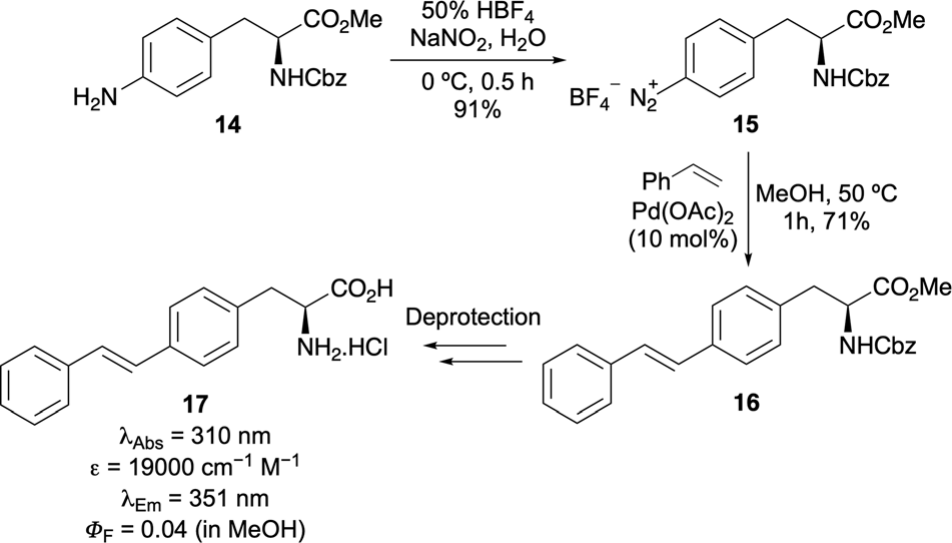
Synthesis of alkyne-extended phenylalanines *via* diazotisation and Heck–Matsuda cross-coupling reaction.

In addition to alkynyl phenylalanines, the conjugation of the aryl unit has been extended through the introduction of alkene spacers.^35^ A two-step methodology comprising of diazotisation of 4-aminophenylalanine derivative 14, followed by a Heck–Matsuda coupling allowed access to analogues bearing cinnamate, vinylsulfone and stilbene side chains. Notably the (*E*)-stillbene-containing compounds (*e.g.*17, Scheme 2) exhibited the most pronounced photophysical properties, characterised by red-shifted absorption and emission spectra compared to phenylalanine. However, the relatively low quantum yields of these compounds due to the flexibility of the chromophore limits their application.

**Fig. 2:**
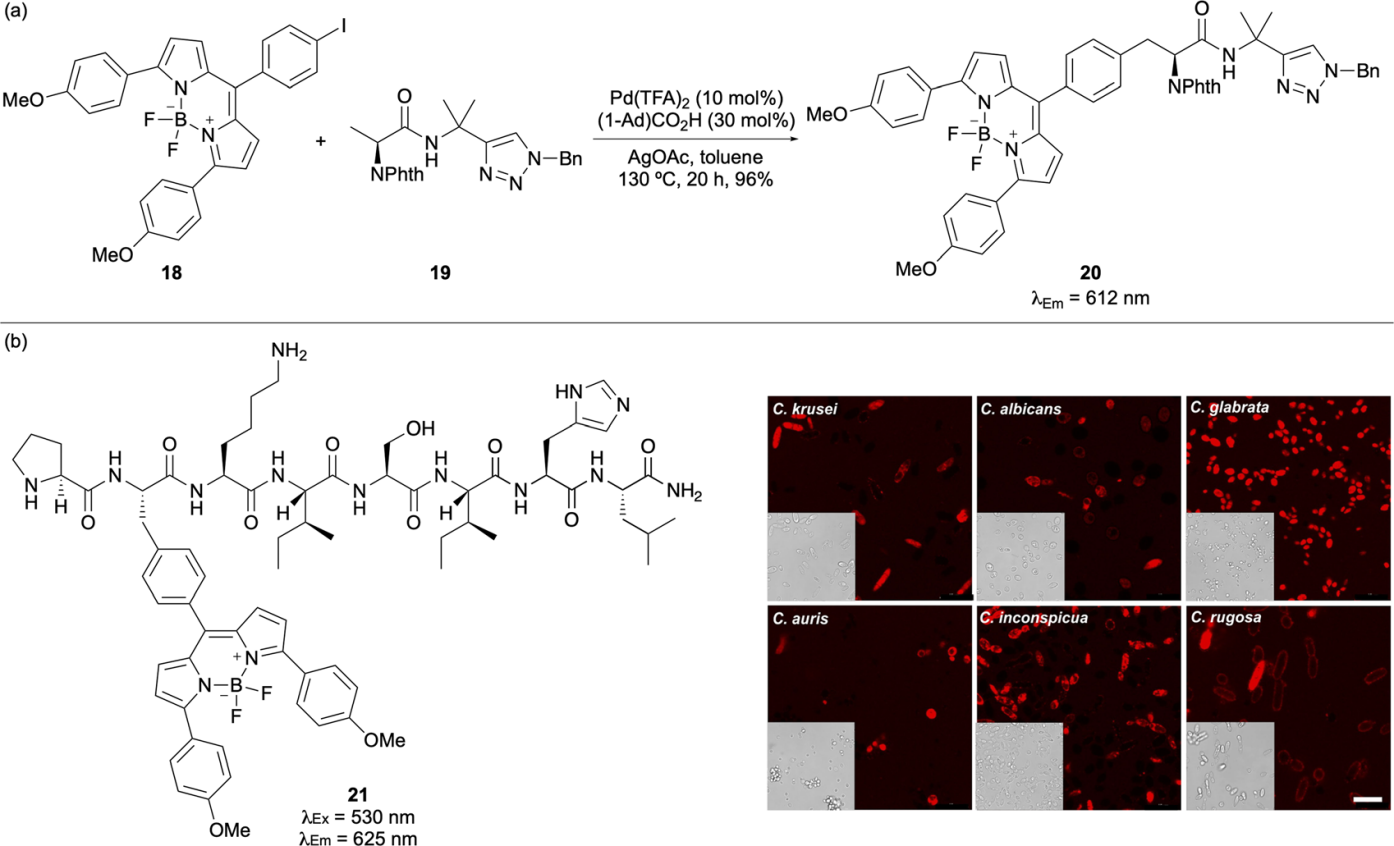
(a) BODIPY labelling of alanine using Pd-catalysed C(sp^3^)–H activation. (b) Incorporation of Phe-BODIPY amino acid into an antimicrobial peptide for labelling of *Candida* cells in urine (scale bars are 10 μm). Cell images reproduced with permission from ref. 37. Copyright 2022, Wiley-VCH Verlag GmbH & Co. KGaA, Weinheim.

Enhanced fluorescent phenylalanine analogues have also been prepared using alternative synthetic strategies. For example, Ackermann and co-workers developed a Pd-catalysed C(sp^3^)–H activation of alanine derivatives for the preparation of BODIPY-labelled phenylalanines (Fig. 2a).^36^ The use of a C-terminal triazole-directing group and 1-adamantanecarboxylic acid [(1-Ad)CO_2_H] as an additive generated adducts in excellent yields and with significantly red-shifted emission. This method was also compatible with BODIPY-labelling of secondary C(sp^3^)–H centres and for the late-stage functionalisation of peptides. In a follow-up study, the Ackermann and Vendrell groups employed computational methods to investigate the energy barrier of the Phe-BODIPY core that facilitates non-radiative decay. The analysis guided the optimal design of Phe-BODIPY adducts with enhanced fluorogenic properties. These adducts were subsequently incorporated into an antifungal peptide (21) and successfully used to detect *Candida* cells in human urine samples (Fig. 2b).^37^ Representing the state-of-the-art in fluorogenic phenylalanines, these Phe-BODIPY adducts are larger than proteinogenic amino acids, but remain relatively compact, enabling their use for important biological applications.

### Modification of tyrosine

**Fig. 3:**
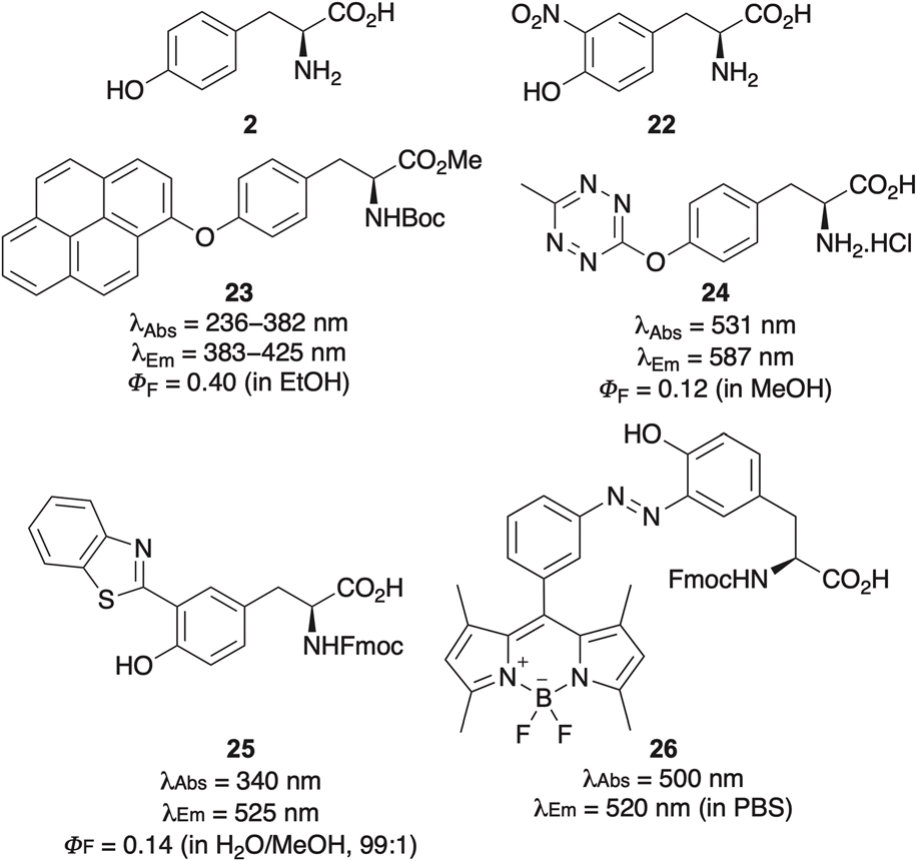
Tyrosine (2), 3-nitrotyrosine (22) and fluorescent structural analogues.

Like phenylalanine, tyrosine (2) possesses intrinsic fluorescence that is significantly limited due to its low quantum yield and susceptibility to photobleaching.^3,13^ The fluorescence of tyrosine is also strongly influenced by the local environment, which limits is use for many imaging applications. Instead, some tyrosine analogues, such as 3-nitrotyrosine (22, Fig. 3) function more effectively as quenchers of other fluorescent compounds due to an ability to engage in electron transfer and energy dissipation processes.^38,39^ Rather than relying on the inherently weak fluorescence of tyrosine, a more effective approach involves covalent modification of its phenolic hydroxyl group with external fluorophores. One such method is the copper-catalysed arylation of tyrosine with aryl bromides, yielding adducts like compound 23, with photophysical properties that are derived from the attached pyrene moiety.^40^ Alternatively, nucleophilic aromatic substitution with bromotetrazines has produced derivatives such as compound 24, which display significantly red-shifted absorption and emission spectra, along with good quantum yields.^41^ The utility of this fluorophore was demonstrated by conjugation of this tyrosine derivative to the peptide drug octreotide, followed by fluorescence quenching *via* an inverse electron-demand Diels–Alder reaction with a strained alkene.

Phenol group functionalisation aside, the construction of fluorescent analogues based on tyrosine remains underexplored, representing an underdeveloped yet promising area in the design of targeted fluorescent probes for protein labelling strategies. Rare examples of fluorophores constructed using the phenol core of tyrosine have proven highly valuable as molecular probes. A notable example is the work by Singh and Verma, who synthesised 3-benzothiazolyl-l-tyrosine 25*via* condensation of 3-formyltyrosine with 2-aminophenothiol (Fig. 3).^42^ This fluorophore exhibited aggregation-induced-emission in water, attributed to the formation of head-to-tail J-aggregates and its utility was demonstrated by the detection of latent human fingerprints on surfaces. Another example was reported by the Lavilla and Vendrell groups who showed adducts such as 26 could be prepared by direct reaction of tyrosine derivatives with diazonium BODIPY salts.^43^ This reaction was applicable for BODIPY insertion within peptides and whole recombinant proteins and, following labelling of immunophilins was used for the detection of the immunosuppressant drug, tacrolimus.

**Scheme 3:**
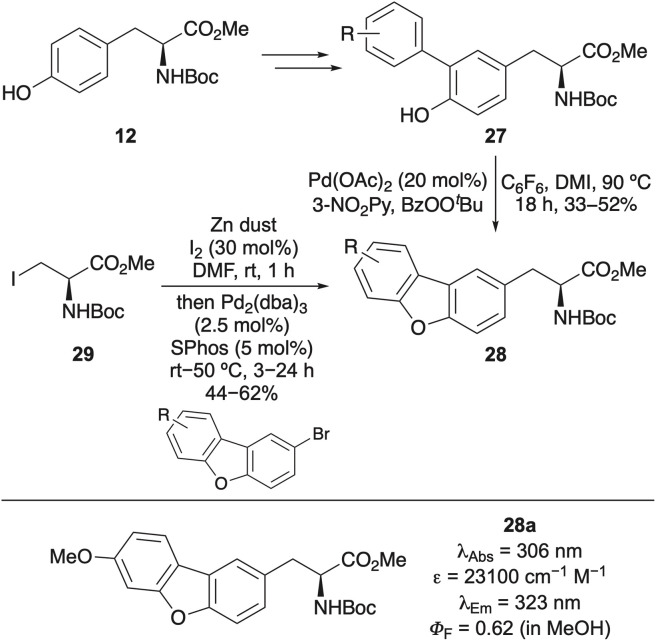
Synthesis of dibenzofuran amino acids, rigid analogues of tyrosine.

Another example is the design and synthesis of dibenzofuran amino acids as extended, rigid structural analogues of tyrosine (Scheme 3).^44^ A small library of these compounds was prepared using two complementary synthetic strategies. The first approach involved bromination of tyrosine derivative 12, followed by Suzuki–Miyaura cross-coupling, yielding 3-aryl tyrosines 27. Cyclisation to the dibenzofuran ring system was then accomplished *via* a palladium(ii)-catalysed C(sp^2^)–H activation and C–O cyclisation. A more efficient and concise route employed Negishi cross-coupling of iodoalanine 29 with brominated dibenzofurans using conditions developed by Jackson and co-workers.^45^ The majority of the resulting dibenzofuran analogues exhibited red-shifted absorption and emission, along with significantly higher quantum yields, attributable to the rigidity of the fluorophore. Methoxy analogue 28a was found to be the brightest tyrosine mimic and incorporated into an internally quenched FRET peptide using SPPS. When paired with 2,4-dinitrolysine as the FRET quencher, dibenzofuran amino acid 28a proved effective in monitoring proteolytic cleavage by trypsin.

**Fig. 4:**
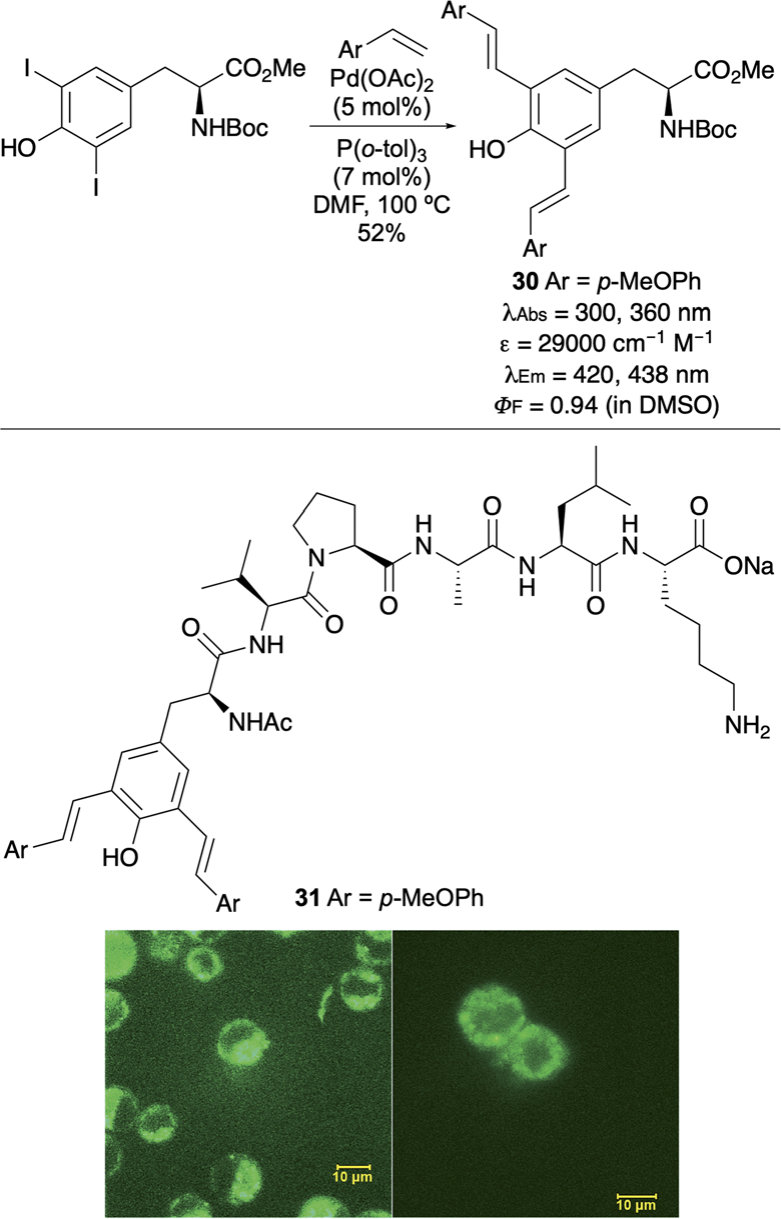
Synthesis of bis-styryl tyrosine 30 and incorporation into a cell penetrating peptide (31) for HeLa (left) and NIH 3T3 (right) cell imaging. Cell images reproduced with permission from ref. 46. Copyright 2015, The Royal Society of Chemistry.

The most comprehensive fluorescent study of tyrosine analogues was conducted by Wang and co-workers, who prepared a series of mono- and di-stilbene adducts *via* palladium-catalysed Heck cross-coupling of iodinated tyrosines with an array of styrene derivatives.^46^ These unnatural amino acids exhibited broad fluorescence emission profiles spanning 400–800 nm, including emission in the near-infrared (NIR) region. Strategic incorporation of pyridine and phenol functionalities enabled pH-dependent red, green and blue emission, with distinct optical signatures in basic, acidic and neutral environments, respectively. Notably, these probes demonstrated fully reversible fluorescent responses to pH and redox changes. The utility of these tyrosine analogues was further validated by incorporation of bis-styryl tyrosine 30 (Fig. 4) into a cell-penetrating peptide (31) *via* SPPS. Confocal fluorescence imaging of HeLa and NIH 3T3 cells confirmed cellular uptake, establishing the application of these amino acids for biological imaging.

### Modification of tryptophan

l-Tryptophan plays an important role in fluorescence-based studies due to its intrinsic fluorescent properties, which stem for the indole side chain.^13^ This natural fluorescence, characterised by sensitivity to local environmental changes has made tryptophan an invaluable internal probe in protein structural and dynamic studies.^3,20^ Researchers have harnessed these properties not only to monitor conformational shifts and protein–protein interactions but also to inspire the development of more sophisticated fluorescent probes.^47^ This has been achieved by exploiting the chemical reactivity of the indole ring to incorporate functional groups with extended conjugation or by replacement of specific atoms. These modifications have produced tryptophan analogues with enhanced quantum yields, red-shifted emission spectra and improved photostability, enabling real-time high-resolution visualisation of cellular process for bioimaging.

**Fig. 5:**
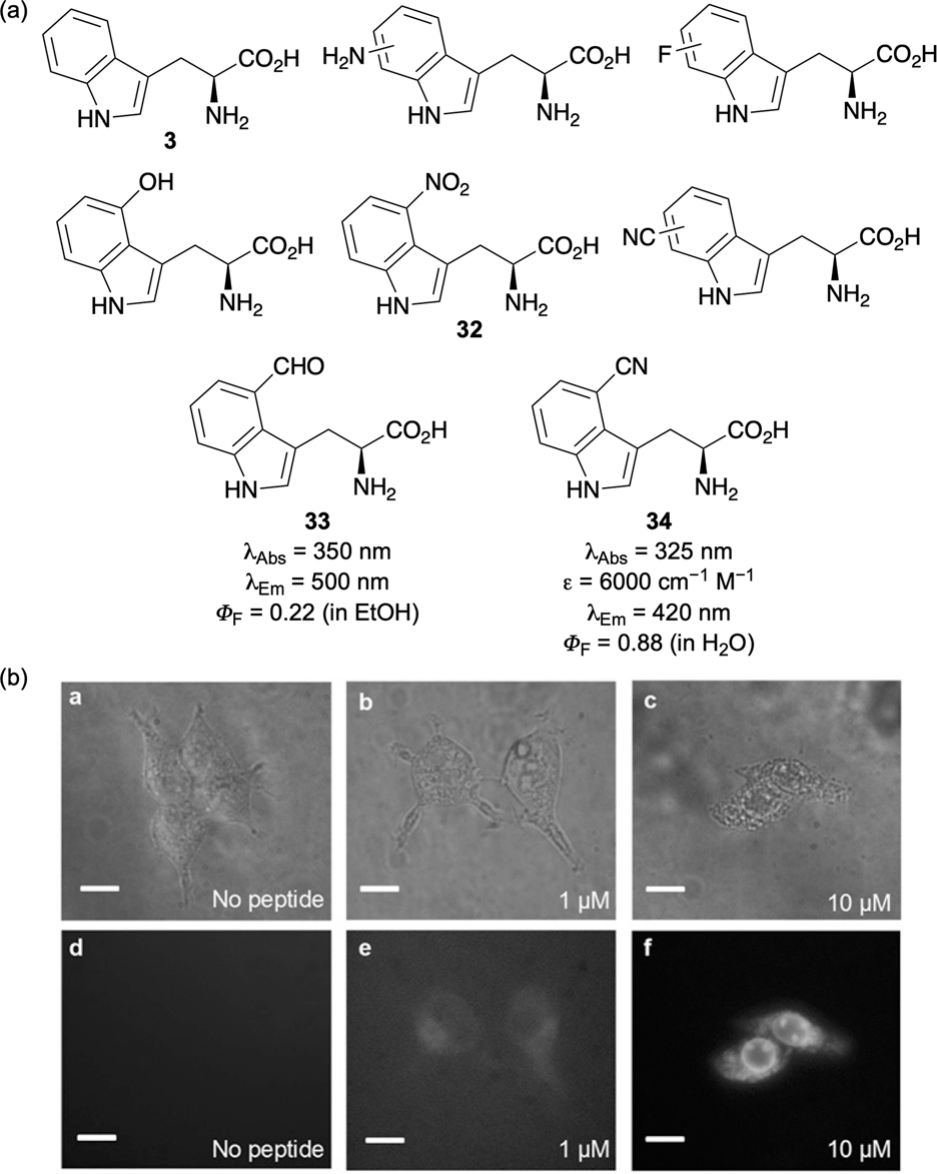
(a) l-Tryptophan and minimally modified structural analogues. (b) Bright-field (a–c) and fluorescent images (d–f) of HEK293T/17 cells using a peptide containing 34 (scale bars are 10 μm). Cell images reproduced with permission from ref. 49. Copyright 2017, The National Academy of Sciences of the USA.

A broad array of minimally modified tryptophan analogues has been prepared incorporating a single functional group (Fig. 5a).^20,47^ Although many of these exhibit modest improvements or in the case of 4-nitro-l-tyrosine (32),^48^ no fluorescence at all, certain purposefully designed charge transfer systems have yielded enhanced properties. For example, the introduction of electron-deficient groups to modulate the electronic transition energy of the indole ring led to the discovery of 4-formyl-l-tryptophan (33), which is excited by visible light, emits around 500 nm and has a good quantum yield of 0.22.^48^ Among these analogues, however, the simplest and most impactful for biological imaging is 4-cyano-l-tryptophan (34).^49^ With red-shifted absorption and emission spectra, good photostability and an excellent quantum yield of 0.88, this fluorescent amino acid has been widely utilised. It has been incorporated into peptides mainly using gene encoding and applied in cell imaging (Fig. 5b),^49^ to study peptide–membrane interactions,^50^ assessment of cell permeability,^51^ as a FRET pair with tryptophan^52^ and for tuning the fluorescent properties of green fluorescent protein (GFP).^53^

**Scheme 4:**
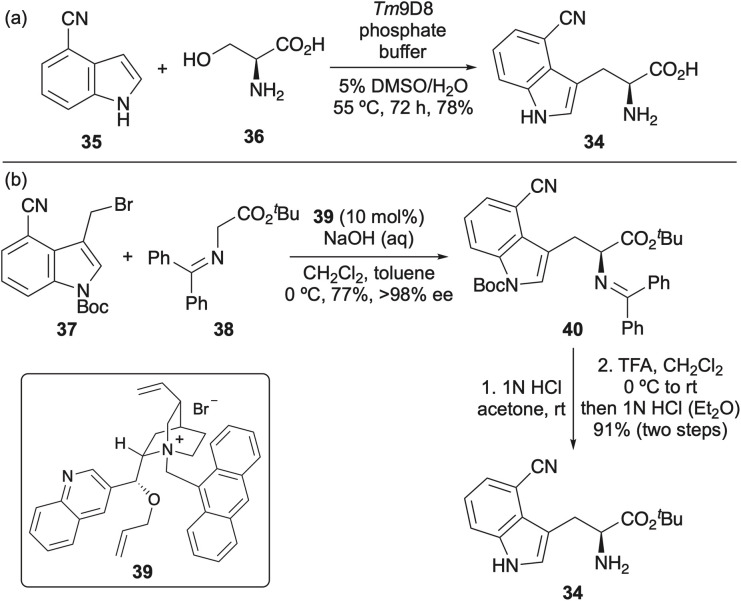
(a) Chemoenzymatic synthesis of 4-cyano-l-tryptophan. (b) Synthesis of 4-cyano-l-tryptophan *via* an enantioselective phase transfer-catalysed alkylation.

Due the importance of 4-cyano-l-tryptophan and the limitations of early syntheses that involved the incorporation of the cyano group *via* moderate yielding cross-coupling methods^49,54^ or enzymatic kinetic resolution of a racemic mixture,^50^ efforts have focused on developing more efficient syntheses to access this compound for biological studies. For example, Arnold and co-workers reported a one-pot chemoenzymatic synthesis from 4-cyanoindole (35) and serine (36) catalysed by tryptophan synthase (TrpB) (Scheme 4a).^55^ Engineering of TrpB from *Thermotoga maritima* generated a variant (*Tm*9D8) that could produce 4-cyano-l-tryptophan (34) in aqueous conditions with a 78% yield. An alternative strategy involved the enantioselective alkylation of glycine-derived imine 38 with cyanoindole bromide 37 in the presence of quinine-based phase transfer catalyst 39 (Scheme 4b).^56^ This method afforded adduct 40 in 77% yield and >98% ee, and was amenable to multi-gram scale (10 g), enabling the efficient preparation of 4-cyano-l-tryptophan (34) or access to the Fmoc-derivative for SPPS.

**Fig. 6:**
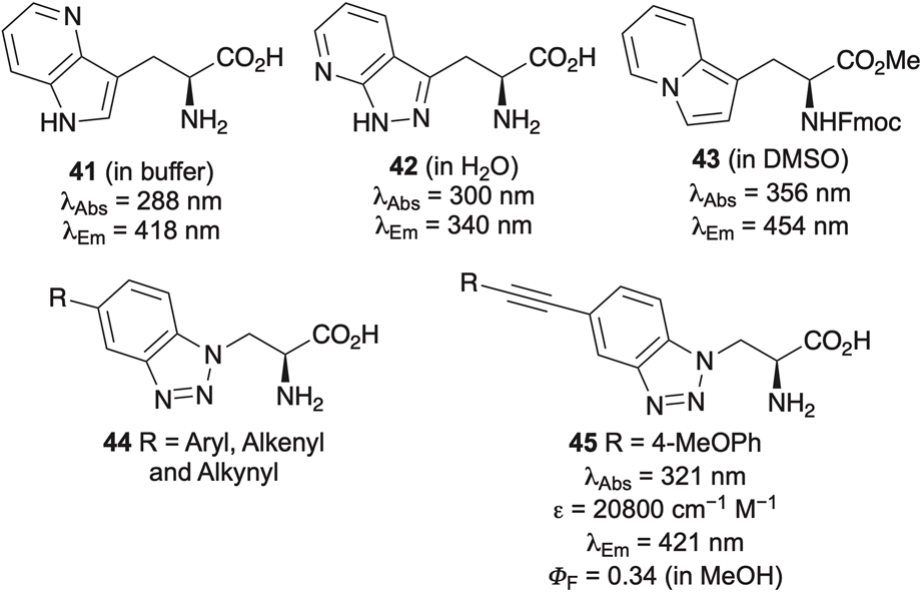
Aza and isostere analogues of tryptophan.

Another modification of tryptophan involving minimal change of the indole ring is the incorporation of additional nitrogen atoms generating azatryptophans (Fig. 6).^20^ Although many azatryptophans exhibit only modest red shifts in absorption and emission wavelengths, they have nonetheless proven valuable for biological studies. For example, 4-aza-l-tryptophan (41) has been genetically encoded into human annexin A5, producing a protein with intrinsic blue fluoresence,^57^ while 2,7-diaza-l-tryptophan (42) has served as a site-specific probe for examining the water environment of tryptophan residues in ribonuclease T1 and used to study the conformational dynamics of asparaginase isozymes.^58^ In contrast to azatryptophans, more pronounced bathochromic shifts in both absorption and emission spectra have been achieved with the tryptophan mimics, indolizinylalanine (43) and the benzotriazole-derived amino acids 44.^59,60^ Notably, benzotriazole analogues featuring C-6 alkynyl substitution, such as compound 45, have emerged as fluorophores with relatively bright emission.^60*b*^

**Fig. 7:**
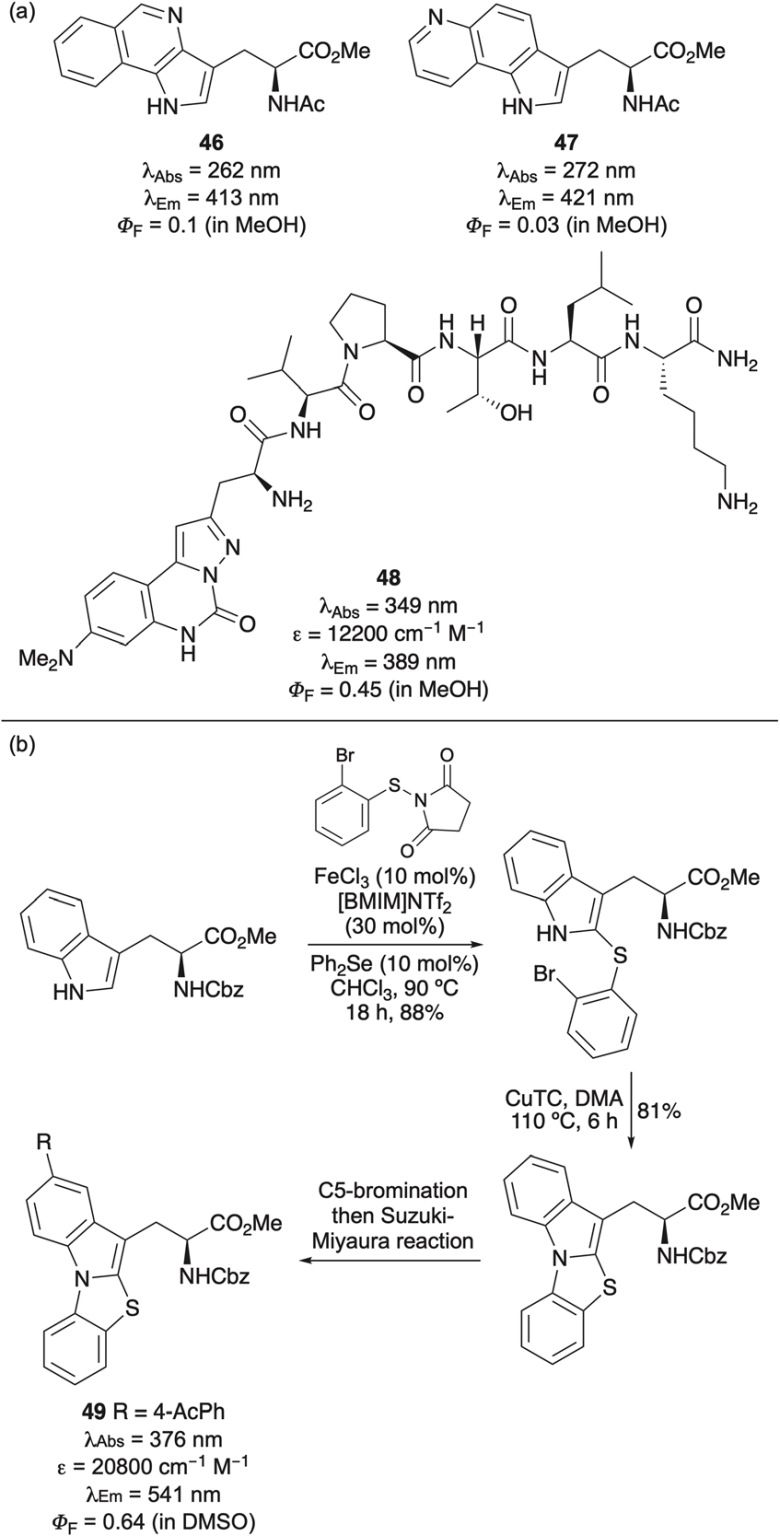
(a) Structurally extended analogues of tryptophan (b) Synthesis and photophysical properties of thiazoloindole amino acids.

Although the introduction of small substituents or additional nitrogen atoms has led to tryptophan analogues with improved fluorescence, achieving more desirable photophysical properties has required more extensive structural modification of the indole ring. Hecht and co-workers demonstrated this approach through the design of tryptophan analogues bearing additional conjugated aromatic rings.^61^ Specifically, amino acids featuring pyrrolo[3,2-*c*]isoquinoline (46) and pyrrolo[2,3-*f*]quinoline (47) side chains were synthesised *via* stereoselective alkylation of the Schöllkopf chiral auxiliary. These analogues exhibited red-shifted emission, large Stokes shifts, high molar absorptivities but lower quantum yields relative to tryptophan (Fig. 7a). The brightest analogue, pyrrolo[3,2-*c*]isoquinoline amino acid 46 was successfully incorporated into dihydrofolate reductase and its fluorescence used to study protein conformational changes.^61*a*^

To enhance the quantum yields of extended tryptophan mimics, a series of rigid pyrazoloquinazoline-derived amino acids was designed and prepared from aspartic acid *via* a multi-step route.^62^ The synthesis involved the formation of a 2-aminoaryl pyrazole side chain, which was cyclised by carbonylation using triphosgene. The resulting analogues exhibited consistently high quantum yields, ranging from 0.42 to 0.56. Notably, the lead dimethylamino derivative proved compatible with two-photon excitation and maintained its fluorescence properties upon incorporation into a cell-penetrating peptide (48) (Fig. 7a).

Other more conjugated tryptophan analogues include the thiazoloindole amino acids, which were designed as rigid, extended fluorophores.^63^ The thiazoloindole core was prepared *via* a dual-catalysed C-2 thioarylation of a tryptophan derivative, followed by copper-mediated intramolecular *N*-arylation (Fig. 7b). Extension of the conjugated system through regioselective bromination and a Suzuki–Miyaura reaction yielded highly fluorescent amino acids with excellent quantum yields, emitting green light. The potential of amino acid 49 for bioimaging applications was further confirmed by excitation *via* two-photon absorption in the near-infrared.

**Fig. 8:**
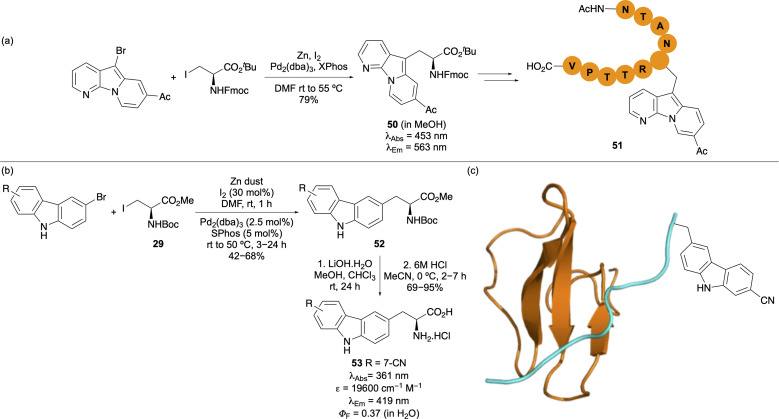
(a) Synthesis of pyrido[3,2-*b*]indolizine amino acid 50 and incorporation into the peptide ligand, Stargazin. (b) Synthesis and photophysical properties of carbazole-derived amino acids. (c) Incorporation of carbazole amino acid 53 into a proline-rich ligand for binding studies with the WW domain protein.

The Jackson method^45^ for amino acid synthesis *via* Negishi cross-coupling has been employed for the synthesis of structurally expanded tryptophan mimics for the study of protein–peptide interactions. Kim, Park and co-workers used this approach to prepare pyrido[3,2-*b*]indolizine amino acid 50, which exhibited significantly red-shifted absorption and emission properties (Fig. 8a).^64^ This amino acid was incorporated into the C-terminal motif of the Stargazin peptide ligand to probe binding with the third PDZ domain of postsynaptic density protein (PSD-95), a key motif in the organisation of signalling proteins. While the resulting peptide 51 showed only modest fluorescent turn-on and low brightness upon binding to the PSD-95 domain, this experiment led to a modified fluorescent peptide that was successfully used for studying this interaction.

Sutherland and co-workers used the Jackson method for the preparation of carbazole amino acids, designed as more conjugated analogues of tryptophan (Fig. 8b).^65^ Their role as tryptophan mimics was validated by substitution into a β-hairpin peptide in place of a tryptophan residue. CD spectroscopy confirmed that the modified peptide retained its β-structure. The environment sensitive 7-cyanocarbazole amino acid 53 was incorporated into a proline-rich peptide ligand *via* SPPS and used to monitor binding to a WW domain protein (Fig. 8c), an interaction found in signalling pathways such as ubiquitination. Fluorescence titration demonstrated that incorporation of amino acid 53 did not disrupt peptide–protein binding, while enabling accurate measurement of the interaction.

**Fig. 9:**
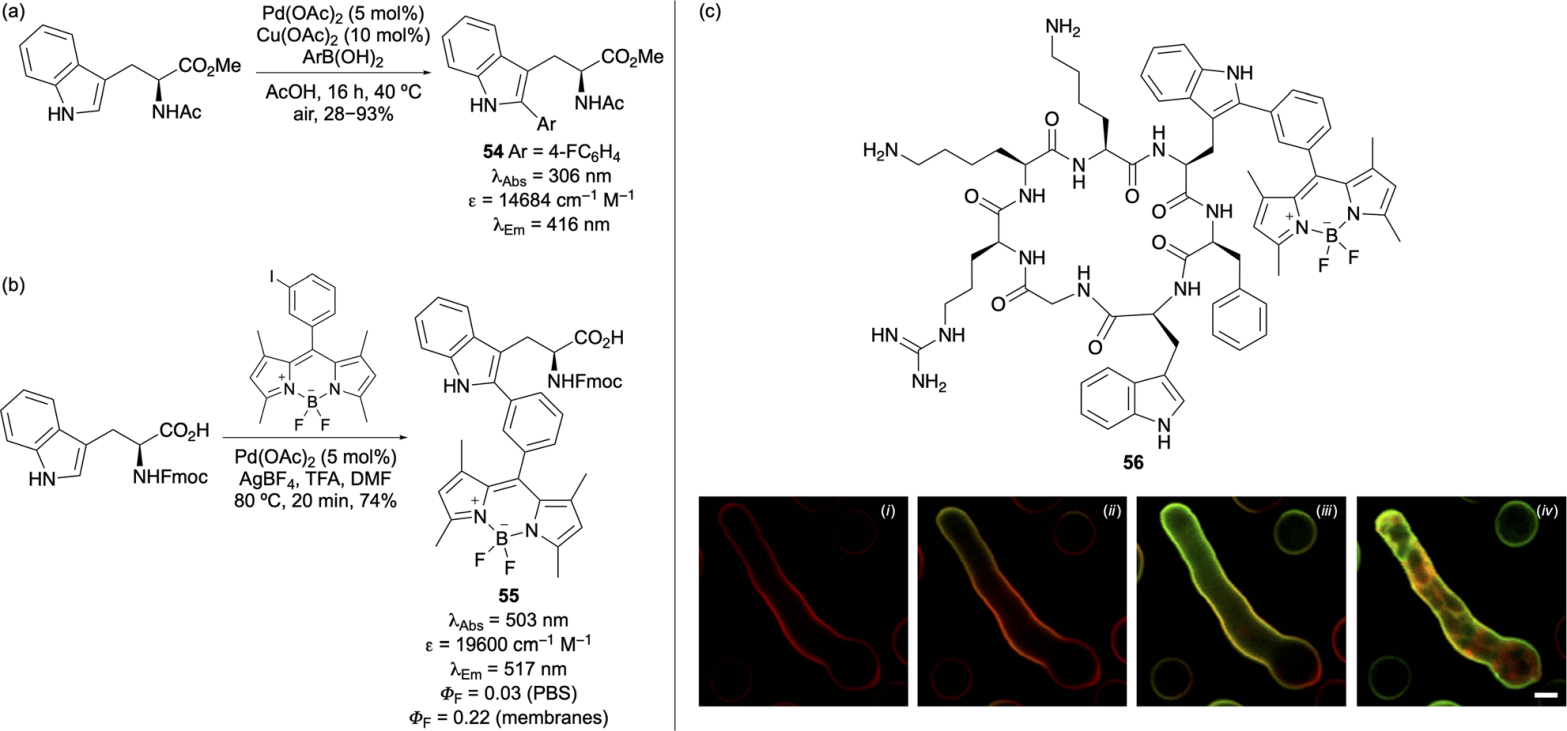
(a) Palladium-catalysed synthesis of 2-aryl tryptophans. (b) Synthesis and photophysical properties of tryptophan-BODIPY adduct 55. (c) Fungal cell imaging using a cyclic peptide containing a tryptophan-BODIPY compound. Panels show 0 min (i), 1 min (ii), 3 min (iii) and 10 min (iv) (scale bars are 2.5 μm). Cell images reproduced with permission from ref. 67. Copyright 2016, Springer Nature Limited.

Another key strategy for enhancing the fluorescent potential of tryptophan involves C2-functionalisation of the indole ring. The inherent reactivity of the C2-position, particularly *via* C(sp^2^)–H activation has enabled the incorporation of diverse functional groups and established chromophores, leading to the development of new probes for biological imaging. Fairlamb and co-workers demonstrated a palladium-catalysed coupling of tryptophan and tryptophan-containing peptides with aryl boronic acids, yielding analogues with red-shifted absorption and emission properties (Fig. 9a).^66^ Vendrell and co-workers showed that tryptophan derivatives could undergo palladium-catalysed C(sp^2^)–H activation and cross-coupling with halogenated BODIPY compounds, producing new amino acids with excellent photophysical properties widely used for imaging applications.^67–69^ For example, tryptophan-BODIPY adduct 55, which exhibits lipophilic-sensitive fluorogenic behaviour (Fig. 9b) was incorporated into cyclic peptide 56.^67^ This cyclic peptide demonstrated high stability in lavage samples and allowed fluorescence imaging of *Aspergillus fumigatus*, showing rapid labelling and accumulation in lipid-rich intracellular compartments (Fig. 9c). Based on these results, multiphoton and lifetime imaging with peptide 56 enabled direct visualisation of *A. fumigatus* in *ex vivo* human tissue.

**Fig. 10:**
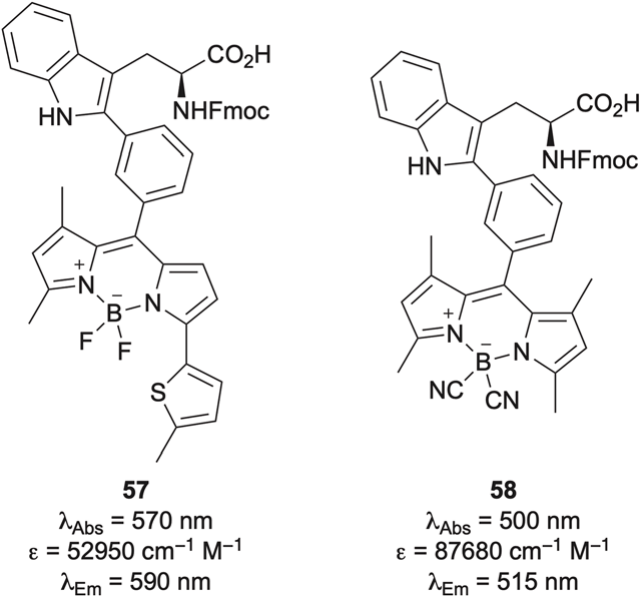
Structures and photophysical properties of tryptophan-BODIPY adducts 57 and 58.

The success of tryptophan-BODIPY adduct 55 has led to the development of next-generation analogues (Fig. 10). Incorporation of a thiophene ring led to amino acid 57, which exhibits red fluorogenic properties.^70^ A cyclic peptide incorporating adduct 57 demonstrated enhanced binding affinity for keratin-1, a protein associated with aggressive breast cancer phenotypes, compared to a similar peptide labelled through lysine-BODIPY conjugation. Subsequent studies confirmed that the cyclic peptide bearing amino acid 57 could be used to image keratin 1 positive cells in intact tumour tissues. Amino acid 57 has also been used to visualise chemotherapy-induced cancer cell death.^71^ Another notable analogue is the cyano-substituted tryptophan-BODIPY adduct 58, which was found to be more acid-resistant, widening SPPS applications.^72^ This amino acid was incorporated into a cyclic peptide and its turn-on fluorescence enabled imaging of GPR54 receptor expression and localisation in human cells.

**Fig. 11:**
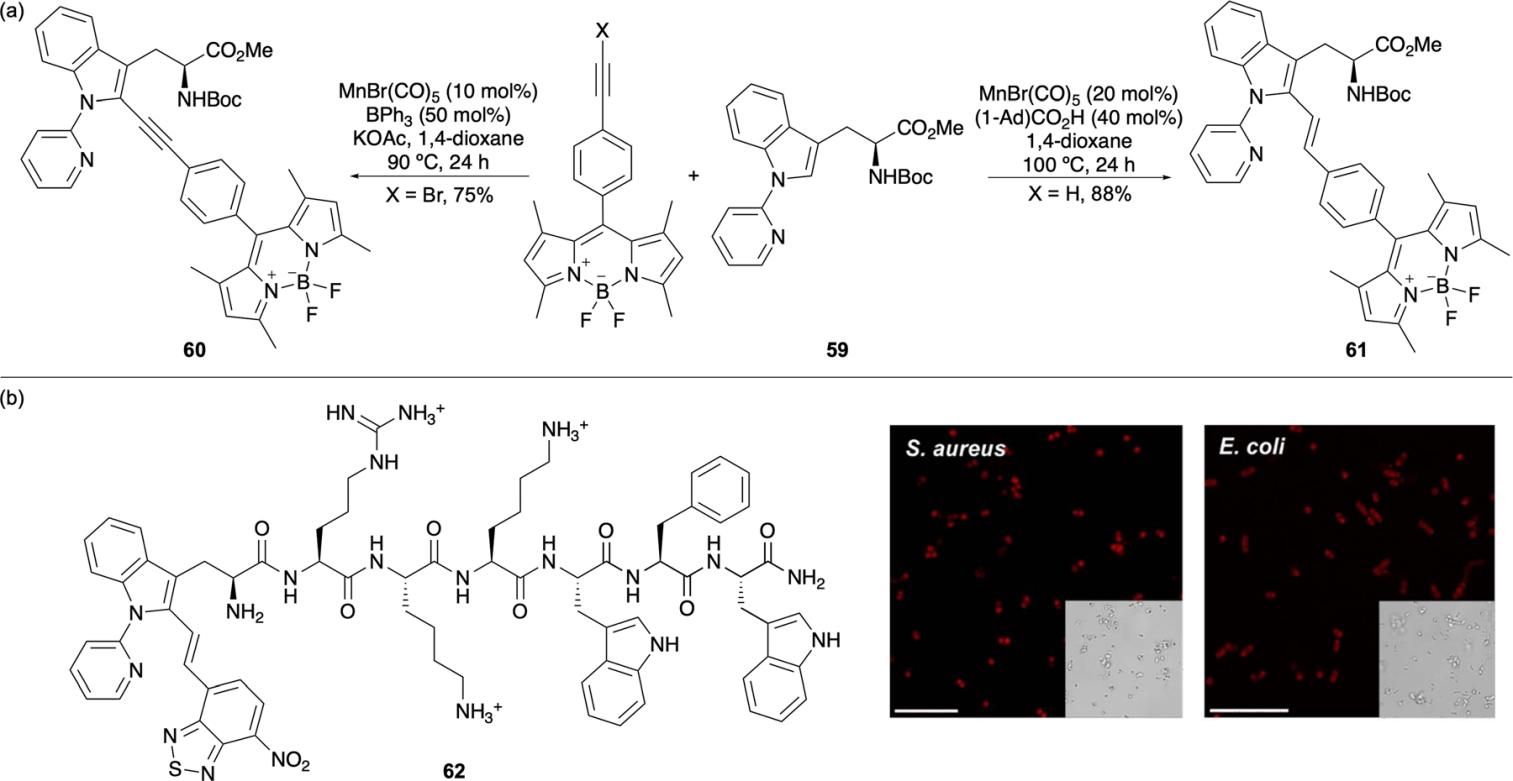
(a) Pyridine-directed, Mn-catalysed synthesis of alkyne- and alkene-linked tryptophan-BODIPY adducts. (b) A nitrobenzothiadiazole-labelled tryptophan peptide (62) used for bacterial cell imaging (scale bars are 10 μm). Cell images reproduced with permission from ref. 77. Copyright 2023, The Royal Society of Chemistry.

The synthesis of novel fluorescent tryptophan analogues *via* metal-catalysed C(sp^2^)–H activation has been expanded to include alkenes and alkynes as coupling partners. Originally developed using ruthenium,^73^ rhodium^74^ and palladium catalysis,^75^ Ackerman, Vendrell and co-workers reported the synthesis of tryptophan-BODIPY derivatives conjugated *via* an alkyne or alkene spacer using pyridine-directed, manganese-catalysed C–H activation (Fig. 11a).^76^ The use of brominated alkynes facilitated an insertion/β-elimination process to access linearly labelled amino acids such as 60. In contrast, the use of terminal alkynes led to an insertion/protodemetalation mechanism to afford bent systems such as 61. Both amino acids showed excellent photophysical properties and further studies revealed a lauric acid derivative of 61 functioned as a fluidity-sensitive probe, displaying increased emission upon binding to cholesterol in cellular membranes.^76^ This feature enabled its application as a turn-on probe for detecting fluctuations in cholesterol levels and activity in live human CD8^+^ T cells.

Pyridine-directed manganese-catalysed C–H activation has also been employed for C2 labelling of tryptophan and tryptophan-containing peptides with nitrobenzodiazole and nitrobenzothiadiazole fluorophores.^77^ The resulting alkene-conjugated derivatives exhibited red shifted absorption (*e.g. λ*_Abs_ = 496 nm) and emission properties (*e.g. λ*_Em_ = 658 nm), along with fluorescence turn-on behaviour in organic media. The compact tryptophan-nitrobenzothiadiazole adduct was incorporated into a bacteria-targeting peptide sequence (62) *via* SPPS and used for wash-free imaging of Gram-positive (*S. aureus*) and Gram-negative (*E. coli*) bacterial cells (Fig. 11b).

Structural modification of the fluorescent proteinogenic amino acids *via* substitution or by incorporation of side chain mimics has significantly expanded their utility as versatile probes in biological imaging. This has yielded new probes with fine-tuned photophysical properties, including red-shifted emission, higher quantum yields, and environmental sensitivities that can be used in the presence of the natural fluorescent amino acids such as tryptophan. Although these modifications increase the size of the amino acid side chain, they still result in relatively compact fluorescent probes that can be readily incorporated into peptides and proteins without severely disrupting native structure or function. These engineered fluorogenic amino acids have been introduced *via* SPPS or genetic code expansion strategies, facilitating real-time imaging of cellular processes, tracking of protein localisation, and monitoring of microenvironmental changes such as pH and polarity. This strategy offers a powerful means to develop high-resolution, minimally invasive fluorescent tools for studying complex biological systems.

## Amino acids with fluorophore-containing side chains

**Fig. 12:**
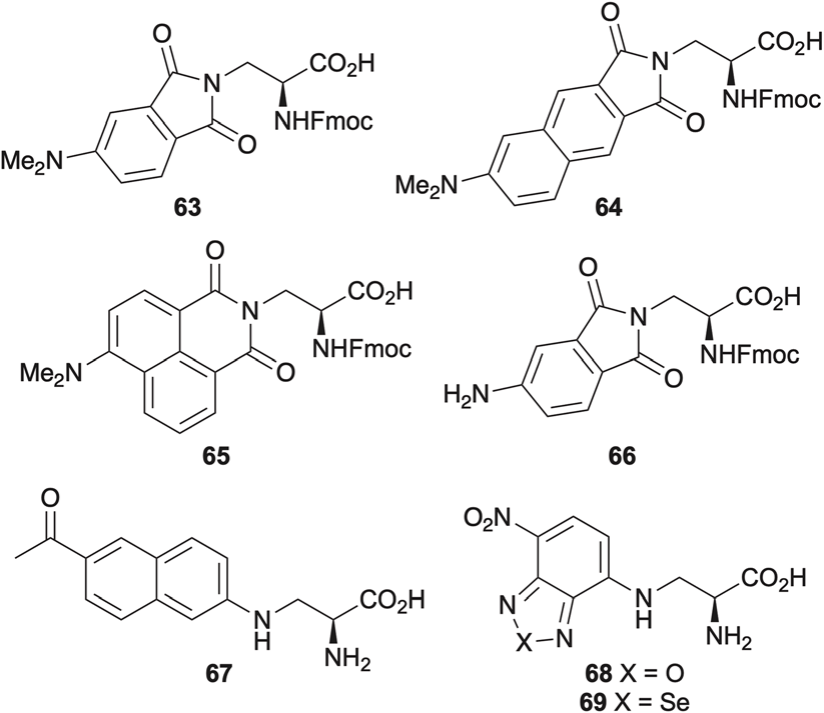
Fluorescent unnatural amino acids derived from 3-aminoalanine.

Another key approach for developing novel small molecule fluorescent amino acids involves the utilisation of an established or designed fluorophore as the amino acid side chain.^15,16^ Although this is often achieved through lysine derivatisation,^78^ creating amino acids comparable in size to proteinogenic residues with the fluorophore embedded within the peptide's secondary structure for localised reporting requires synthetic building blocks with shorter side chains. A common approach involves 3-aminoalanine with fluorophore incorporation *via* the 3-amino side chain. This strategy was used by Imperiali and co-workers who developed a series of environment sensitive fluorescent amino acids derived from 3-aminoalanine incorporating dimethylamino-substituted phthalimide (63),^79^ 2,3-naphthalimide (64)^80^ and 1,8-naphthalimide (65) moieties (Fig. 12).^81^ These amino acids could be excited above 350 nm and were found to emit at wavelengths of >500 nm, allowing their use for the measurement of various protein–protein interactions. More recently, these and structurally related analogues (66), have been employed as probes for transmembrane peptide localisation^82^ and as turn-on sensors for the cancer protein target, proliferating cell nuclear antigen (PCNA).^83^

l-3-(6-Acetylnaphthalen-2-ylamino)-2-aminopropanoic acid (l-Anap) (67) with a 3-aminoalanine core was developed as a charge-transfer probe, exhibiting a shift in emission from 490 nm in water to 420 nm in ethyl acetate (Fig. 12).^84,85^ This amino acid was amenable to genetic encoding and incorporated into yeast^84^ and mammalian cells,^86,87^ to probe structural changes caused by ligand binding and localisation of proteins using both one- and two-photon excitation. l-Anap 67 has also been incorporated into Bax, a proapoptotic protein and used as a FRET pair with the Hsp70-YFP fusion protein, to study how this interaction is affected by organelle-targeting substances.^88^

3-Aminoalanine has also been used to generate nitrobenzodiazole (NBD) derivatives *via* nucleophilic aromatic substitution of halogenated 4-nitrobenzodiazole compounds. Amino acids such as 68, which exhibits red-shifted absorption and environment-sensitive emission have been used to monitor proteolysis and peptide conjugation to glass surfaces for application in micorarrays (Fig. 12).^89,90^

Vendrell and co-workers synthesised various NBD analogues and identified red-emitting (*λ*_Em_ = 601 nm) and photostable benzoselenadiazole amino acid 69 for wash-free microscopy applications.^91^ This small amino acid could be incorporated into peptides using SPPS with retention of structure and binding properties and used for imaging postsynaptic density protein-95 nanoclusters in mouse brain samples.

**Fig. 13:**
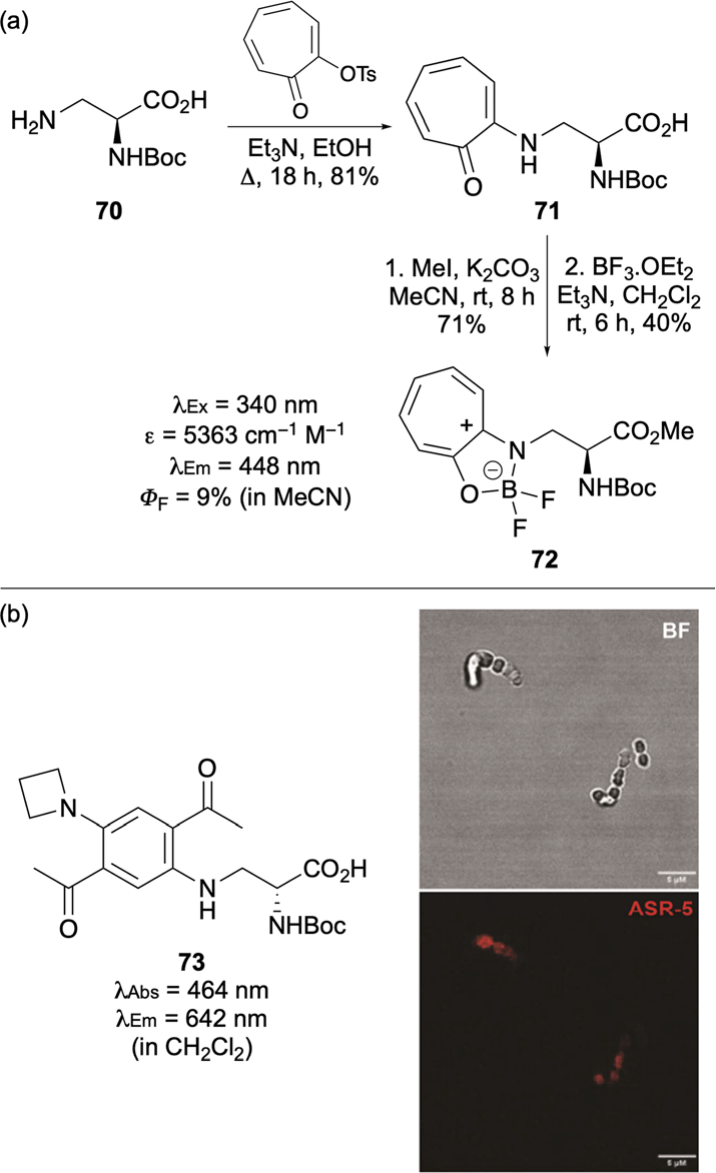
(a) Synthesis and fluorescent properties of troponyl-derived amino acids. (b) Structure of single-benzene-based amino acid 73 and application for bacterial cell imaging (scale bars are 5 μm). Cell images reproduced with permission from ref. 93. Copyright 2025, American Chemical Society.

Novel BODIPY-like amino acids have been prepared *via* coupling of 3-aminoalanine with a tosylated derivative of tropolone (Fig. 13a).^92^ Esterification and reaction with boron trifluoride produced borondifluoro derivative 72, which exhibited red-shifted photophysical properties and internalisation into HeLa cells with low cytotoxicity.

Quantum chemical calculations and structure activity relationship analysis has led to the discovery of small single benzene fluorophores with lipid-sensitive emission in the near infrared (NIR).^93^ Amino acid analogues of these were prepared using a microwave-assisted one-pot palladium-catalysed cross-coupling of dibrominated bis-acetophenones with both azetidine and 3-aminoalanine. The resulting d-amino acid 73 was used as a d-alanine mimic for wash-free fluorescent imaging of peptidoglycan biosynthesis in *Enterococcus faecium* bacterial cells (Fig. 13b).^93,94^

**Scheme 5:**
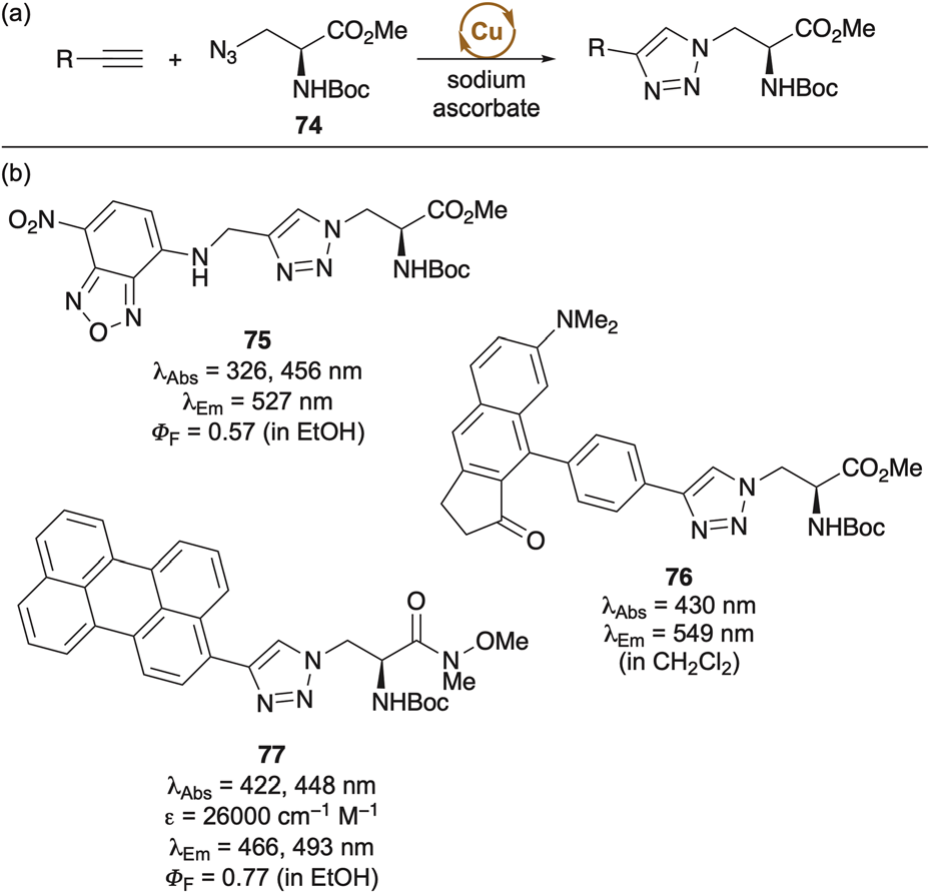
(a) Incorporation of fluorophores using copper-catalysed click reaction. (b) Selected triazole linked fluorescent amino acids.

The compact and structurally related 3-azidoalanine (*e.g.*74) has also been used as an amino acid synthetic building block to incorporate fluorophores.^82,95–99^ Copper-catalysed click reactions with alkynes containing polyaromatic and bespoke fluorophores have led to efficient synthesis of triazole linked amino acids with excellent photophysical properties and high quantum yields (Scheme 5). These compounds have been used for various applications; for example, the charge-transfer perylene-linked amino acid 77, demonstrated enhanced fluorescence upon binding to bovine serum albumin (BSA), enabling its use as a sensor.^98^ While this approach has proven effective for the synthesis of single residue fluorophores, the use of Cu(i) and ascorbate can promote the oxidation of histidine and arginine residues within peptides. Additionally, the cell toxicity of copper limits *in vivo* applications.^100^ These challenges, however, can be overcome by employing strain-promoted azide–alkyne cycloaddition (SPAAC) reactions, which proceed without the need for copper catalysis.^101^

**Fig. 14:**
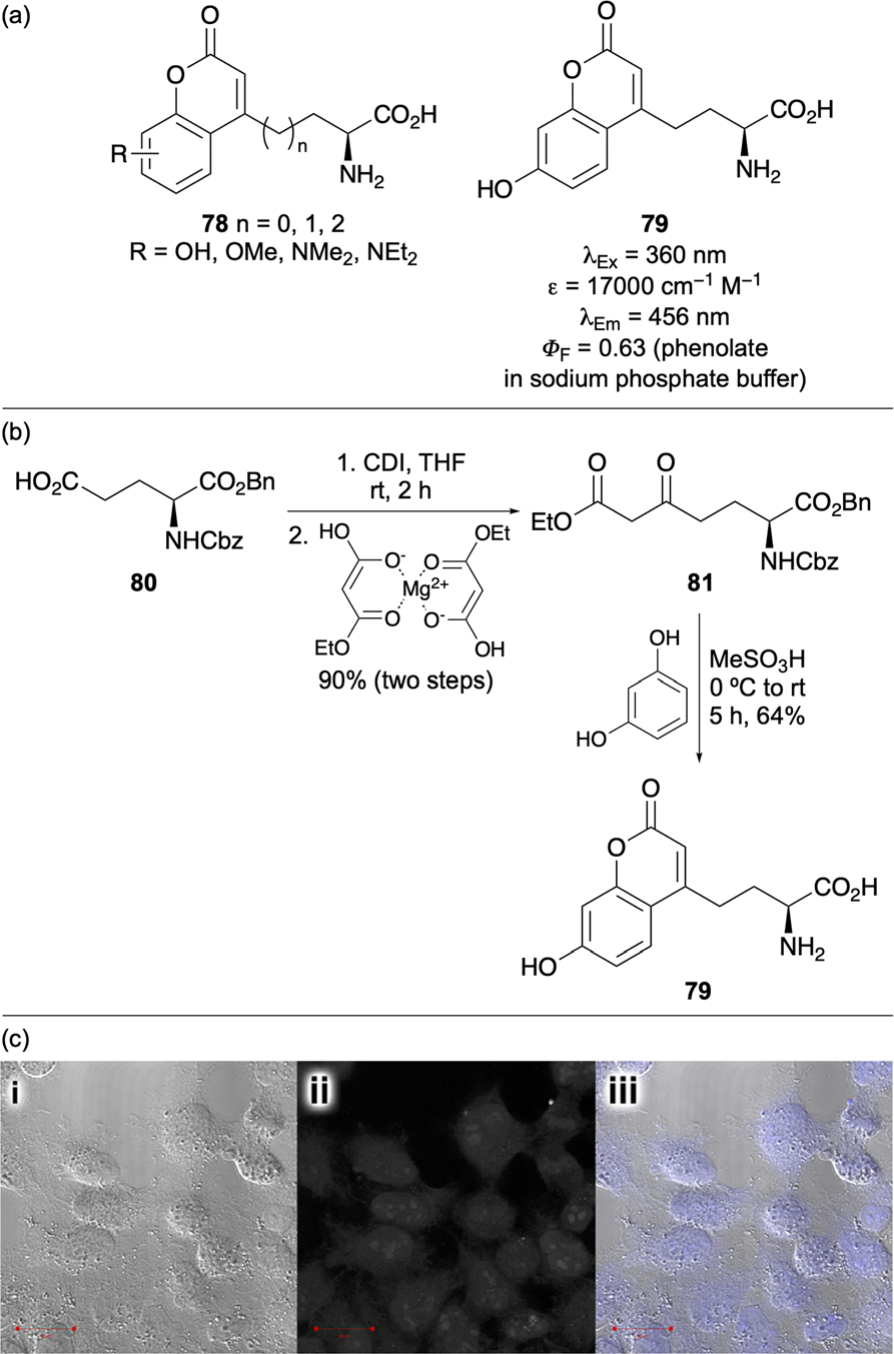
(a) Coumarin-derived fluorescent amino acid probes. (b) Optimised and scalable synthesis of l-(7-hydroxycoumarin-4-yl)ethylglycine (79). (c) Images of HeLa cells using HIV-Tat peptide with amino acid 79 positioned at the C-terminus. Cell images reproduced with permission from ref. 106. Copyright 2013, Elsevier.

Another key strategy for developing novel fluorescent amino acids involves the design of new synthetic methodologies that enable fluorophore construction directly within the amino acid side chain. This approach has been successfully used to incorporate a variety of established fluorophores, resulting in probes with broad applications in sensing and imaging. A common approach includes amino acids bearing coumarin-based side chains.^102^ Due to the compact structure of the 2*H*-chromen-2-one motif, its strong fluorescence and large Stokes shift, these biocompatible residues have been widely employed for biological imaging. Coumarins bearing electron-donating substituents (*e.g.*78) conjugated to the electron-deficient enone have been developed as environment-sensitive, charge transfer probes (Fig. 14a). Access to these compounds either involve attachment of the intact coumarin to a racemic or chiral glycine equivalent^103^ or by stepwise synthesis of the chromen-2-one using a suitably modified amino acid from the chiral pool.^104^ This latter approach *via* a von Pechmann reaction enabled access to l-(7-hydroxycoumarin-4-yl)ethylglycine (79), a polarity and pH-sensitive fluorescent amino acid first genetically encoded in *E. coli* by Schultz and co-workers.^105^ The Martin group later reported an optimised and scalable synthesis of this important probe.^106^ They showed that activation of glutamic acid derivative 80 using carbonyldiimidazole, followed by reaction with freshly prepared ethyl magnesium malonate allowed the efficient preparation of the corresponding β-keto ester 81 (Fig. 14b). Subsequent von Pechmann cyclisation with resorcinol in neat MeSO_3_H afforded the coumarin core, while simultaneously deprotecting the amino and carboxylic acid groups. This route enabled the multigram-scale preparation of amino acid 79, facilitating its use in SPPS. The amino acid was incorporated at various positions within the Leu-enkephalin pentapeptide to demonstrate pH-sensitive fluorescence. It was also introduced at different sites within the cell-penetrating HIV-Tat peptide for HeLa cell imaging (Fig. 14c). The accessibility of amino acid 79 has supported its application in numerous studies. Genetically encoded into proteins and peptides, it has been used to measure conformational dynamics of DNA replication^107^ and investigate protein–ligand binding,^108^ such as that of biotin with streptavidin^109^ and of fatty acids with fatty acid-binding protein.^110^

**Fig. 15:**
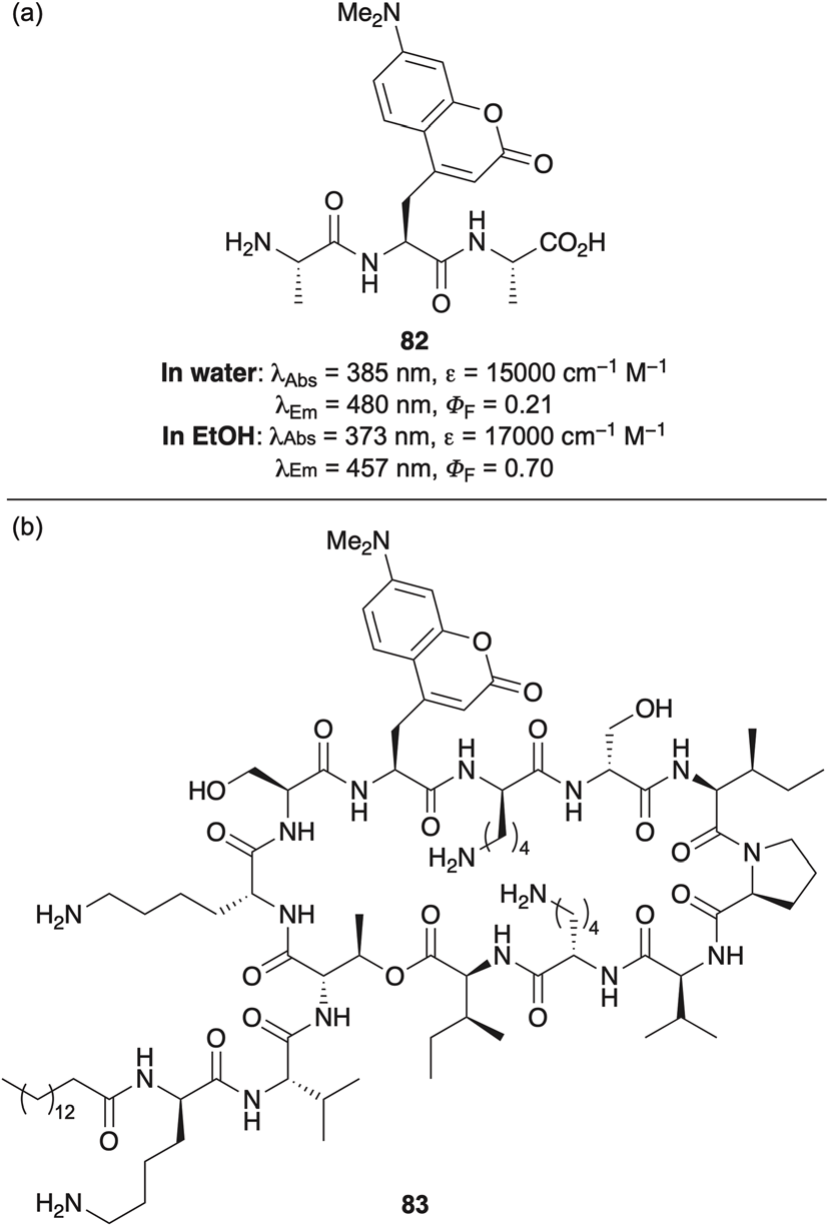
(a) Structure and photophysical properties of 7-dimethylaminocoumarin amino acid containing tripeptide 82. (b) Paenibacterin analogue 83 containing 7-dimethylaminocoumarin amino acid.

Amino-substituted coumarin amino acids also possess environment-sensitive fluorescent properties, making these valuable tools for probing biological interactions. Noden and Taylor developed an enantioselective synthesis of 7-dimethylaminocoumarin-4-alanine *via* alkylation of a chiral nickel(ii) glycine Schiff base complex, producing the amino acid with 97% ee.^111^ The photophysical properties of the resulting amino acid were examined within a tripeptide (82), in which fluorescence intensity increased in less polar environments (Fig. 15a). These environment-dependent properties were exploited by incorporation of the amino acid into an analogue of paenibacterin (83), a broad spectrum cyclic lipodepsipeptide antibiotic and employed to monitor interactions with model liposomes, lipopolysaccharides and live bacteria (Fig. 15b).

**Fig. 16:**
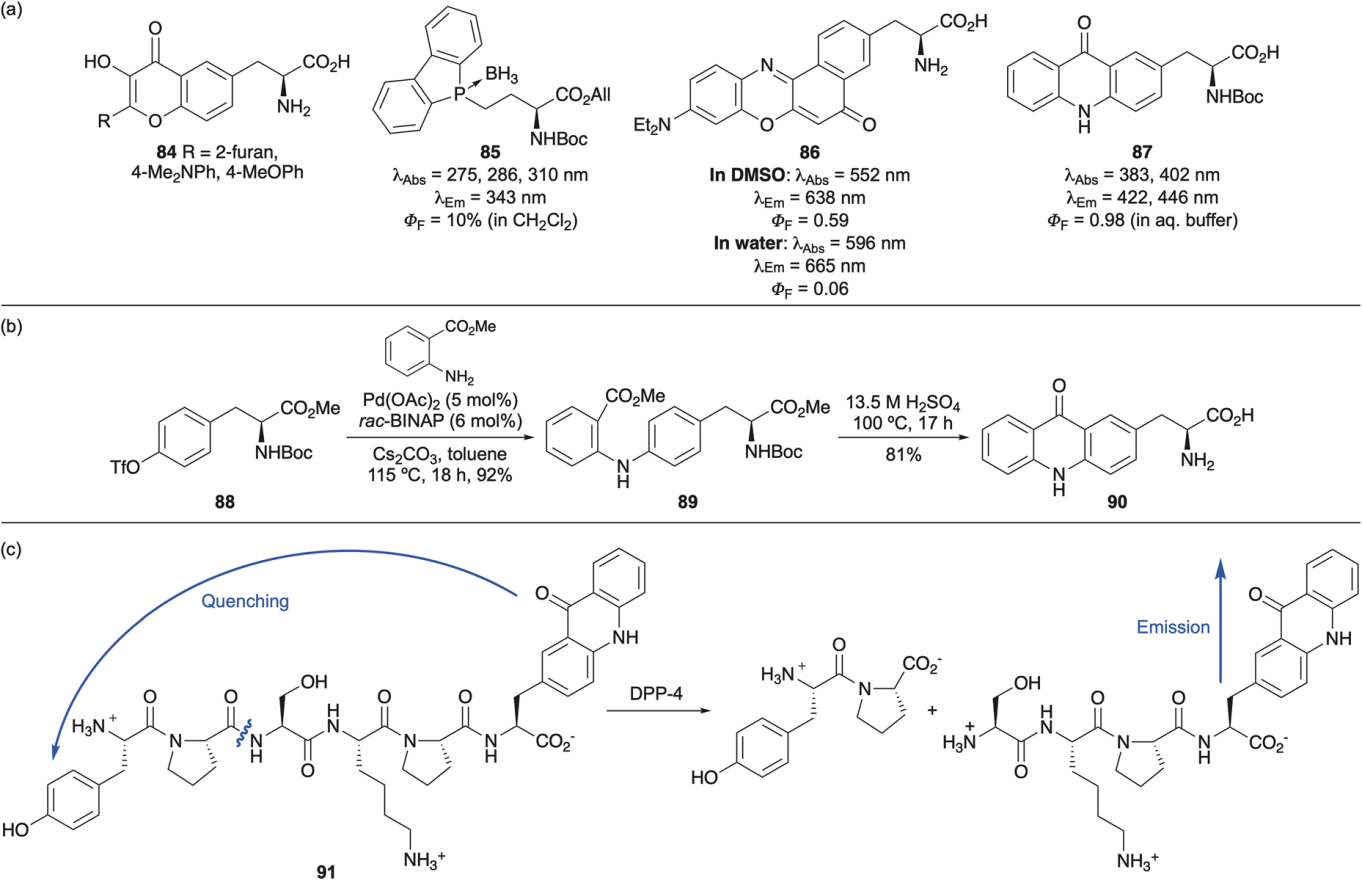
(a) Various fluorescent amino acids synthesised *via* a chiral pool approach. (b) Multi-gram synthesis of acridonylalanine 90. (c) Use of acridonylalanine as part of a FRET peptide to monitor DPP-4 activity.

The chiral pool approach to install fluorophore side chains from proteinogenic amino acid derivatives has been used to access a diverse range of structures (Fig. 16a). Mely and co-workers employed tyrosine to access flavone-derived amino acids 84.^112^ These exhibit dual emission fluorescence *via* excited state intramolecular proton transfer and on incorporation into peptides, were used to probe peptide-nucleic acid interactions and to monitor penetration into cell membranes. Naturally occurring amino acids were also used for the preparation of phospholyl(borane) amino acids (*e.g.*85) and solvatochromic Nile Red derivative 86.^113,114^ Due to the red-shifted properties of 86, peptide derivatives were used for *in vitro* characterisation of biomolecular interactions with proteins and plasma membrane, as well as for cellular ratiometric confocal imaging.

The chiral pool approach has also been employed for the preparation of acridonylalanine (87) (Fig. 16a), a fluorescent amino acid valued for its high quantum yield and long fluorescent lifetime (*τ* = 15.7 ns) under aqueous conditions.^115,116^ A recent multi-gram synthesis of acridonylalanine used a Buchwald–Hartwig coupling of triflate-activated tyrosine derivative 88 with methyl 2-aminobenzoate (Fig. 16b).^117^ Acid-mediated cyclisation and concurrent deprotection gave the parent amino acid in excellent overall yield and moderate stereoselectivity (4 : 1, l/d). Petersson and co-workers have incorporated acridonylalanine into proteins and peptides *via* gene encoding or SPPS and used the resulting probes to investigate the dynamics of protein–protein interactions^118^ and for fluorescence lifetime imaging microscopy (FLIM).^119^ In FILM, acridonylalanine outperformed endogenous fluorophores and engineered fluorescent proteins (*e.g.* GFP), owing to its longer fluorescent lifetime.^119^ Additionally, acridonylalanine has been widely used as a FRET donor in studies of peptide unfolding,^116^ small molecule-induced conformational changes^120^ and protease sensing.^117^ In the latter case, it was incorporated into a short peptide (91) using SPPS, with a N-terminal tyrosine acting as a quencher (Fig. 16c).^117^ This construct was subsequently used to measure the activity of dipeptidylpeptidase 4 (DPP-4), a protease involved in regulating glucagon-like peptide 1 (GLP-1) levels in the bloodstream. Further applications of acridonylalanine (87) include the measurement of protein conformational changes using transition metal ion FRET.^121^ In addition, the success of 87 as a biological imaging tool has led to the development of a wide range of structural analogues,^122^ which have been used as improved FRET donors for peptide cleavage assays^123^ or as sensitisers of Eu(iii)-binding proteins.^124^

**Fig. 17:**
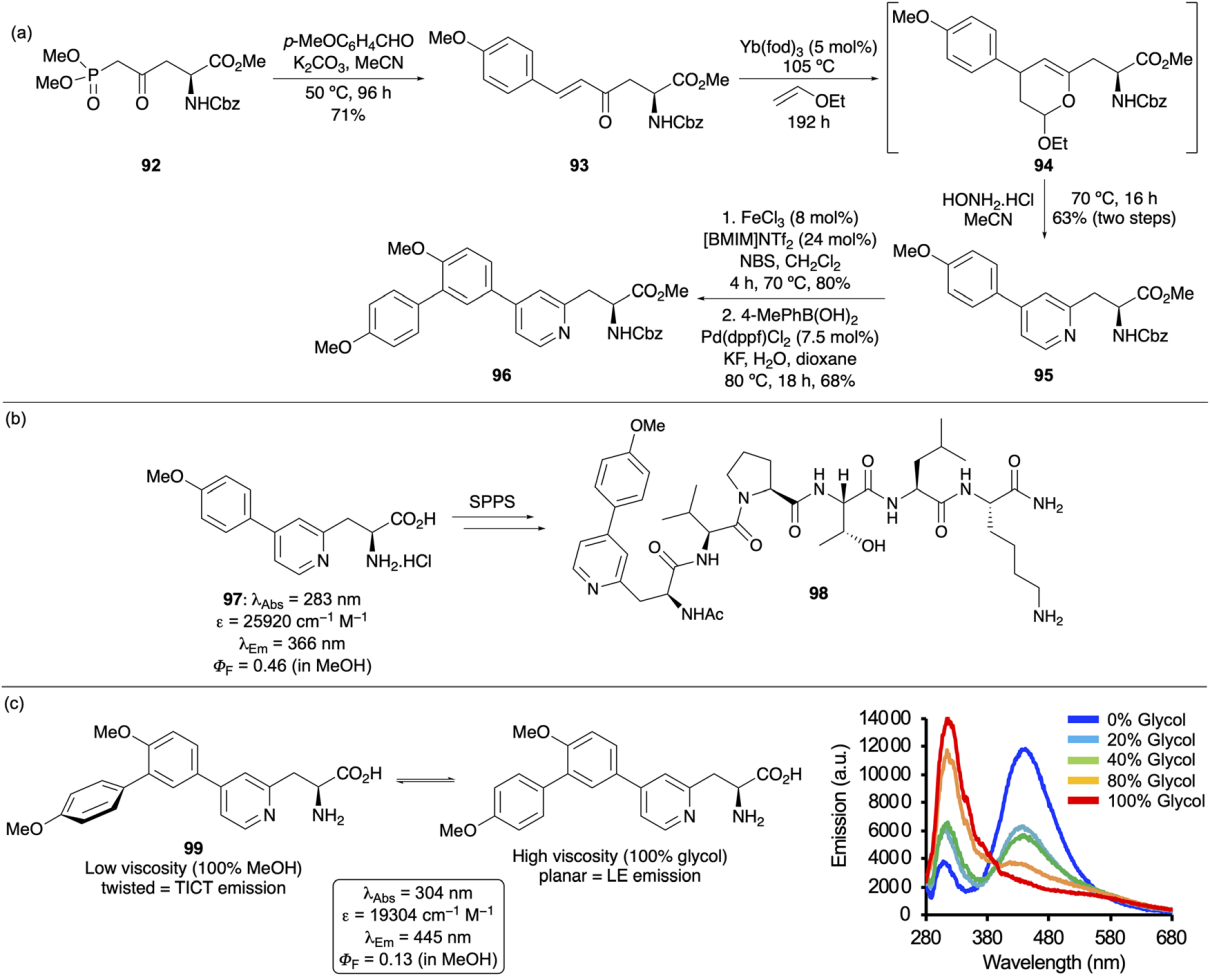
(a) Synthesis of biaryl and triaryl pyridine-derived amino acids. (b) Photophysical properties of amino acid 97 and incorporation into a cell penetrating peptide. (c) Photophysical properties and viscosity sensitivity of amino acid 99.

Aspartic acid has been used as a building block to access amino acids with ynone and enone side chains.^125^ These functionalities were subjected to cycloaddition reactions, resulting in the preparation of fluorescent charge-transfer based amino acids with pyridine^126,127^ and pyrimidine-containing side chains.^128^ In the case of pyridine-containing amino acids, these were prepared in two steps from the corresponding enone-derived amino acid (Fig. 17a).^126,127^ Following a Horner–Wadsworth–Emmons reaction of a phosphonate ester obtained from aspartic acid, resulting enone 93 was subjected to a regioselective ytterbium-catalysed hetero-Diels–Alder reaction with ethyl vinyl ether. Knoevenagel–Stobbe reaction of resulting dihydropyran 94 with hydroxylamine yielded the biaryl pyridine-derived amino acid 95. From this series, the charge transfer-based *p*-methoxyphenyl analogue 97 showed the best photophysical properties (*Φ*_F_ = 0.46) and was SPPS compatible with incorporation into a cell-penetrating peptide (98) (Fig. 17b).^126^ The conjugation of these fluorophores was extended by iron(iii)-catalysed bromination, followed by a Suzuki–Miyaura reaction, to form biaryl-substituted pyridines such as 96 (Fig. 17a). As well as red-shifted absorption and emission, these triaryl amino acids (*e.g.*99) were found to have viscosity sensitive fluorescence based on aryl group conformation (Fig. 17c).^127^ At low viscosity (100% MeOH, 0% glycol), the fluorophore was found to adopt a twisted conformation, resulting in twisted intramolecular charge transfer (TICT) emission at 445 nm. Under higher viscosity conditions, through the addition of increasing amounts of ethylene glycol, amino acid 99 was found to adopt a more planar conformation, resulting in emission from the locally excited (LE) state (315 nm). Thus, polyaromatic amino acids such as 99 have potential as fluorescent viscosity sensors for biological applications.

As these examples demonstrate, the direct incorporation of fluorophores to generate unnatural amino acids with side chains markedly different from those of proteinogenic residues has yielded probes with improved properties that can still be effectively integrated into peptides for imaging applications. However, given the wide array of known fluorophores yet to be explored using this approach and the continuing advances in synthetic methodologies, such as metal-catalysed cross-coupling and C–H activation reactions that enable direct side-chain installation in a single step—this strategy for creating novel fluorescent amino acids is yet to be fully exploited.

## Summary and outlook

Over the last decade, advances in the design and synthesis of unnatural amino acids have led to the development of environment sensitive fluorescent probes. These probes offer a powerful alternative to traditional approaches that rely on extrinsic fluorophores, such as bulky fluorescent proteins or dyes conjugated *via* linkers, methods that often risk disrupting native protein structure or function. In contrast, fluorescent amino acids, introduced through gene encoding or SPPS, serve as intrinsic labels that better preserve biological integrity and functionality.

As highlighted by this perspective, a key strategy to access useful peptidic probes has involved the modification of the three naturally fluorescent amino acids, tryptophan, phenylalanine and tyrosine. As the amino acid with the strongest fluorescence, research has focused on the modification of tryptophan. This has yielded a diverse set of analogues with enhanced photophysical properties that have been used in applications ranging from the measurement of protein–protein interactions^65^ to detecting fungal infections in human tissue.^67^ Although useful analogues of phenylalanine have also been discovered, particularly as probes for lipophilic-rich environments,^34,37^ the discovery and development of new fluorescent tyrosine compounds remains underexplored. Given its electron rich phenol group and prevalence in many key proteins, the development of fluorescent tyrosine analogues represents a promising but underutilised strategy, especially for the design of pH and polarity-sensitive probes.

Another complementary approach involves the incorporation of established or bespoke fluorophores directly into amino acid side chains. Structures based on phthalimides,^79–81^ coumarins^102–111^ and flavins,^112^ have expanded the functional range of peptide-embedded fluorophores, enabling properties such as dual emission^112,129^ and viscosity-sensitive fluorescence *via* conformational control.^127^ These molecular innovations suggest a much broader landscape of future applications, although these remain to be fully realised.

Despite the exciting advances in the field, challenges remain. Although fluorescent unnatural amino acids can be site-specifically introduced into proteins through various strategies, the choice of incorporation site is critical. Numerous studies have demonstrated that optimal positioning is key to preserving the protein's structural integrity and function while enhancing the photophysical properties of the fluorescent amino acid.^28,29,106,109,112,130^ To address this, machine learning models are now being explored to predict optimal incorporation sites.^131^ Further challenges relate to the size of fluorescent unnatural amino acids. The small size required for compatibility with peptide scaffolds often limits the photophysical performance of fluorescent amino acids, resulting in UV or visible-range excitation that is suboptimal for *in vivo* imaging due to poor tissue penetration and high background autofluorescence. Two-photon excitation offers a partial solution by enabling longer-wavelength activation,^62,63,112,132^ offering deeper tissue penetration and reduced phototoxicity. Nonetheless, the requirement for specialised equipment and the limited two-photon cross-section of many small fluorophores remain technical barriers. To broaden biological applicability, especially in live-cell or *in vivo* contexts, additional fluorescent amino acids that can be efficiently excited at wavelengths above 400 nm *via* one-photon methods are still needed. Achieving this will depend on the design of highly conjugated yet compact side chains—structures that could unlock a new generation of probes for biological, medical, and imaging applications.

## Author contributions

The manuscript was written through contributions of both authors. Both authors have given approval to the final version of the manuscript.

## Conflicts of interest

There are no conflicts to declare.

## Data Availability

No primary research results, software or code have been included and no new data were generated or analysed as part of this review.
